# Copy Number Gains of VPS72 Drive De Novo Lipogenesis and Hepatocarcinogenesis via ATF3/mTORC1/SREBP1 Axis

**DOI:** 10.1002/advs.202411368

**Published:** 2025-04-30

**Authors:** Qinglin Zhang, Yunxing Huang, Yin Tong, Kenneth Tsz Chun Ng, Jiangwen Zhang

**Affiliations:** ^1^ School of Biological Sciences The University of Hong Kong Hong Kong SAR 999077 China; ^2^ Department of Pathology School of Clinical Medicine The University of Hong Kong Queen Mary Hospital Pokfulam Hong Kong SAR 999077 China; ^3^ Centre for Oncology and Immunology Hong Kong Science Park Hong Kong SAR 999077 China

**Keywords:** epigenetics regulation, lipid metabolism, mTORC1 signaling

## Abstract

Hepatocellular carcinoma (HCC) is the predominant form of primary liver cancer and a major contributor to cancer‐related mortality globally. Central to its pathogenesis is the dysregulation of lipid metabolism in hepatocytes, leading to abnormal lipid accumulation. Our bioinformatics analysis has identified the histone acetyltransferase complex subunit VPS72 as being associated with HCC, yet the precise molecular mechanisms through which VPS72 contributes to hepatocarcinogenesis remain poorly understood. Our analysis of extensive HCC patient cohorts identifies a significant proportion with VPS72 copy number gains, which are strongly linked to adverse prognostic outcomes. By integrating RNA‐Seq, ChIP‐Seq, ATAC‐seq, and experimental validation, we show that VPS72 overexpression activates mTORC1 signaling, subsequently promoting lipid synthesis and driving HCC progression. We further uncover that VPS72 modulates the epigenetic landscape by enhancing DNA methylation at the ATF3 promoter, resulting in ATF3 repression and subsequent activation of mTORC1. This study elucidates a novel regulatory axis that links dysregulated lipid metabolism with HCC progression, highlighting potential epigenetic and metabolic targets for therapeutic intervention.

## Graphical abstract

VPS72 overexpression activates mTORC1 signaling, subsequently promoting lipid synthesis and driving HCC progression. VPS72 modulates the epigenetic landscape of ATF3 promoter, such as H2A.Z acetylation reduction and DNA methylation increase, which results in ATF3 repression and subsequent activation of mTORC1. These findings provide new insights into the epigenetic regulation of ATF3 by VPS72 and its implications for HCC progression and therapeutic targeting.

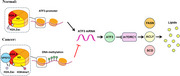

## 1 Introduction

Hepatocellular carcinoma (HCC) is the most prevalent type of primary liver cancer in adults, representing the sixth leading cause of cancer‐related mortality in the United States and the fourth most commonly diagnosed cancer worldwide in 2022.^[^ ^1^ ^]^ Despite the adoption of treatments such as resection, liver transplantation, and transarterial chemoembolization for early‐to‐intermediate stage HCC, there remains no highly effective treatment for late‐stage HCC, resulting in poor prognosis for these patients.^[^ ^2^ ^]^ Therefore, elucidating the molecular mechanisms underlying HCC progression and developing novel therapeutic strategies is of urgent necessity.

The liver is the central organ controlling lipid homeostasis, including lipogenesis and lipid oxidation. Dysregulated hepatic lipid metabolism results in lipid droplet accumulation in hepatocytes, contributing to conditions such as obesity, diabetes, fatty liver disease, and liver cancer.^[^ ^3^, ^4^ ^]^ In liver cancer cells, fatty acid synthesis is elevated, and excess lipid droplets serve as a fuel source for unlimited growth and invasion.^[^ ^5^, ^6^ ^]^ Beyond fatty acid uptake, hepatocytes convert glucose into fatty acids through de novo lipogenesis during the fed state. Key enzymes in lipogenesis, including stearoyl‐CoA desaturase (SCD), ATP citrate lyase (ACLY), and fatty acid synthase (FASN), are found upregulated in HCC,^[^ ^7^ ^]^ and are regulated by sterol response element‐binding protein (SREBP, gene name SREBF) family.^[^ ^8^ ^]^ Besides, the mTORC1 (mechanistic target of rapamycin complex 1) signaling pathway is crucial in promoting lipogenesis by regulating the expression of numerous lipogenic genes. mTORC1 enhances hepatic lipogenesis by activating the trafficking, processing, and transcription of SREBPs through multiple pathways.^[^ ^9^, ^10^, ^11^ ^]^ Given the pivotal role of mTORC1 in lipid synthesis, uncovering novel pathways related to mTORC1 could provide valuable insights into the connection between aberrant lipogenesis and HCC development.

VPS72, also referred to as YL1, serves as a common component in the mammalian TIP60 histone acetyltransferase complex of the INO80 family and the SRCAP (SWI2/SNF2‐related CBP activator protein) complex.^[^ ^12^ ^]^ VPS72 functions as a histone chaperone, interacting with the H2A.Z‐H2B dimer and incorporating it into nucleosomes.^[^ ^13^, ^14^, ^15^ ^]^ Given that the TIP60 and SRCAP complexes modulate the genome‐wide distribution of H2A.Z, aberrant VPS72 levels are thought to have a broad impact on the epigenetic landscape in HCC. In 2019, our study was the first to report the oncogenic potential of VPS72 in HCC development.^[^ ^16^ ^]^ Despite prior research, the contribution of VPS72 to HCC development remains incompletely characterized. To fill this gap, we explored the oncogenic function of VPS72 in HCC through comprehensive patient data analysis and both in vitro and in vivo assays. By combining bioinformatic analyses with experimental validation, we demonstrate that VPS72 activates mTORC1 signaling, thereby promoting lipid synthesis and driving HCC progression. Mechanistically, we found that at the epigenetic level, VPS72 increases methylation at the promoter region of ATF3, a well‐established transcription factor and tumor suppressor, leading to the downregulation of ATF3, subsequent activation of mTORC1 signaling, and enhanced lipogenesis. Our findings elucidate the complex role of VPS72 as an epigenetic regulator of lipid metabolism in HCC and establish a critical link between the development of cancer and lipid homeostasis.

## 2 Results

### 2.1 VPS72 Exerts Oncogenic Role in HCC Progression

**Figure 1:**
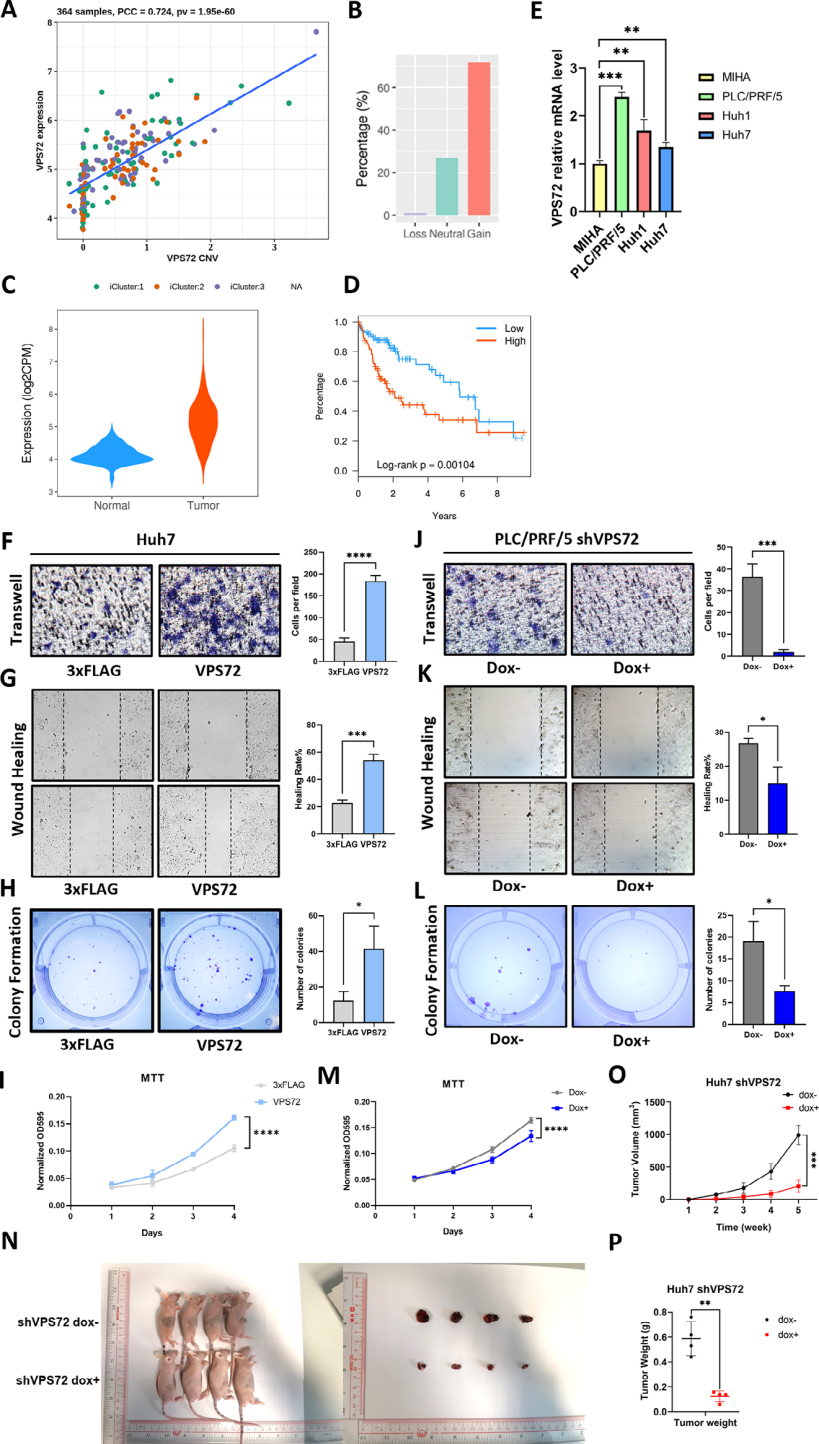
VPS72 promoted migration and proliferation of HCC cells in vitro and tumor growth in vivo. A) Correlation of the copy number and mRNA level of VPS72 in LIHC patients. B) Proportion of VPS72 loss, neutral, and gain in LIHC patients. C) mRNA expression level of VPS72 in LIHC patients. D) Survival of LIHC patients grouped by the expression level of VPS72. Figure A–D was generated with our previous work CR2Cancer webserver. E) The basal VPS72 expression levels in HCC cell lines (Huh7, PLC/PRF/5, and Huh1) and the normal hepatocyte cell line MIHA. Migration was examined by F) Transwell and G) Wound healing assay, photo taken at 0 h (upper) and 24 h (lower). And the proliferation was tested by H) Colony formation and I) MTT assay. J) Transwell of VPS72‐knockdown PLC/PRF/5 cell line and K) Wound healing assay, photo taken at 0 h (upper) and 24 h (lower). And the proliferation was tested by L) Colony formation and M) MTT assay. N) Photograph of mice and tumor in the mouse xenograft assay. O) Tumor volume and P) Tumor weight. *n* = 3 independent experiments for (f–m); *n* = 4 subjects per group in (n).

Based on initial evidence linking VPS72 to hepatocellular carcinoma progression, we examined TCGA Liver Hepatocellular Carcinoma (LIHC) patient data to explore the correlation between VPS72 copy number, expression levels, and patient survival outcomes. A significant positive correlation was identified between VPS72 copy number and its expression (**Figure** **1**A), suggesting that early oncogenic amplification of VPS72 may drive its overexpression and contribute to HCC development. The VPS72 gene was found to be present in extra copies in over half of the samples (Figure 1B), indicating that VPS72 copy number gains may play a pivotal role in HCC pathogenesis. VPS72 expression was markedly higher in tumor tissues compared to normal controls (Figure 1C), and survival analysis revealed a strong association between elevated VPS72 expression and reduced overall survival (Figure 1D). Stratified analysis of VPS72 expression in HCC driven by alcohol consumption, hepatitis B virus (HBV), hepatitis C virus (HCV), and non‐alcoholic steatohepatitis (NASH) indicated significant upregulation of VPS72 across all subtypes compared to normal tissues. However, VPS72 expression was comparatively lower in NASH‐driven HCC than in alcohol‐, HBV‐, or HCV‐driven cases (Figure S1, Supporting Information).

We next examined basal VPS72 expression in HCC cell lines (Huh7, PLC/PRF/5, and Huh1) and the normal hepatocyte cell line MIHA using qPCR. VPS72 expression was consistently higher in all HCC cell lines compared to MIHA, with PLC/PRF/5 exhibiting the highest expression and Huh7 the lowest. Based on these findings, Huh7 was selected for VPS72 overexpression experiments, while PLC/PRF/5 was used for knockdown in vitro studies (Figure 1E). For in vivo experiments, Huh7 cells were chosen due to their moderate tumor growth rate and the favorable health condition of the mice used in the study.

To validate the oncogenic potential of VPS72 in HCC cells, we conducted overexpression and knockdown experiments in Huh7 and PLC/PRF/5 cell lines, respectively. Overexpression of VPS72‐3xFLAG fusion protein, compared to the control group overexpressing the 3xFLAG epitope tag, resulted in enhanced migration ability of Huh7 cells, as demonstrated by transwell and wound‐healing assays (Figure 1F,G). Additionally, VPS72 overexpression accelerated cell proliferation, as shown by MTT and colony formation assays (Figure 1H,I). Enhanced migration and proliferation were also observed in normal liver LO2 cells upon VPS72 overexpression (Figure S2A, Supporting Information).

Conversely, knockdown of VPS72 using a doxycycline‐inducible lentiviral system suppressed the migration and proliferation abilities of PLC/PRF/5 cells (Figure 1J–M). Similar suppression of migration was observed in HepG2 cells following VPS72 knockdown (Figure S2B, Supporting Information). To further substantiate the findings, the knockdown of H2AFZ (encoding H2A.Z) was performed in PLC/PRF/5 cells as a reference. The migration and proliferation abilities were inhibited upon doxycycline treatment (Figure S3, Supporting Information), consistent with the known oncogenic role of H2A.Z in HCC as reported by previous studies.^[^ ^17^, ^18^ ^]^ We also constructed VPS72‐knockdown Huh7 cell line and adopted it to the nude mice tumorigenicity assay. The result indicates that compared with the control group, VPS72 knockdown significantly represses tumor growth in vivo (Figure 1N–P). Taken together, these results demonstrate that VPS72 acts as an oncogene in HCC development.

### 2.2 VPS72 Overexpression Mainly Represses Gene Expression in HCC Cell Line

**Figure 2:**
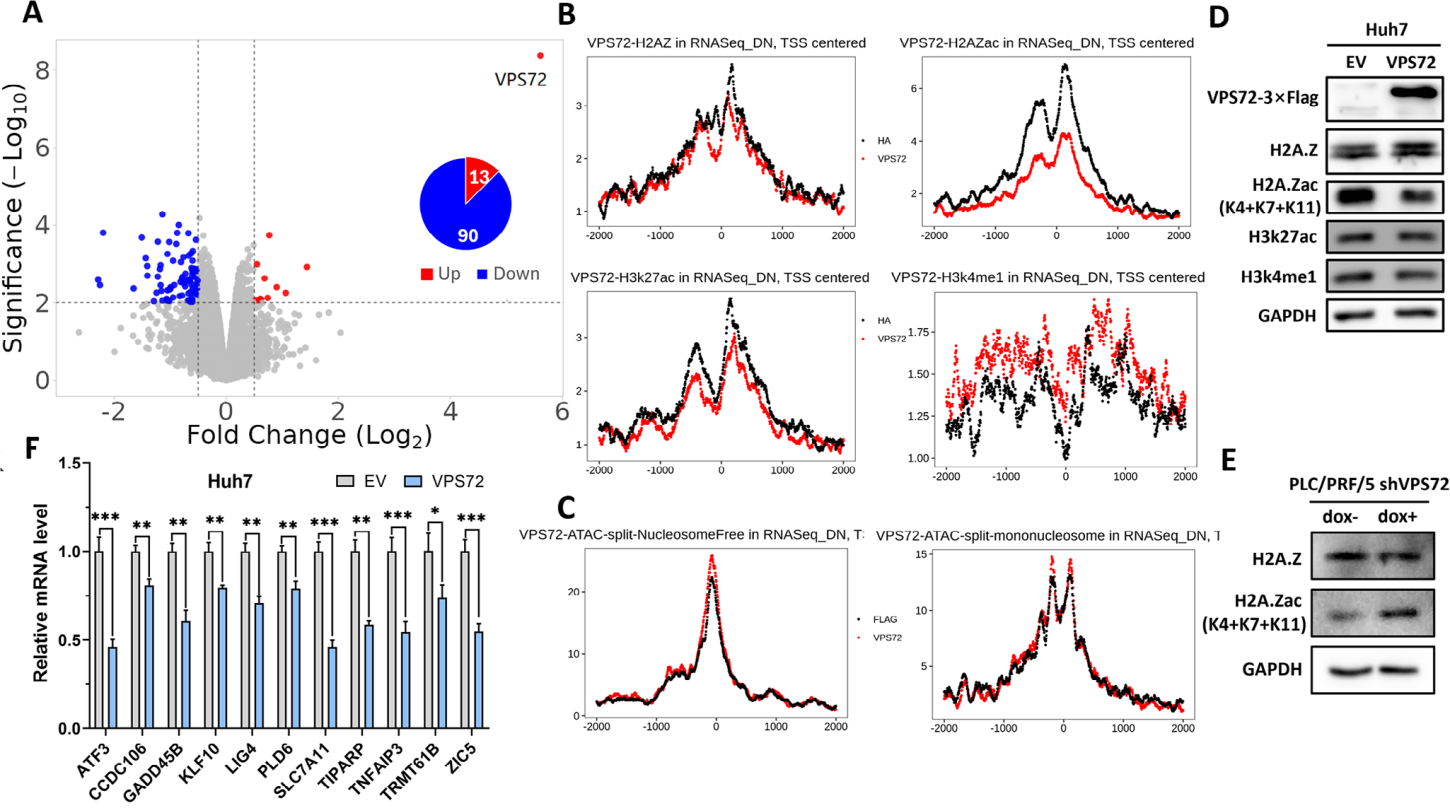
VPS72 overexpression mainly repressed gene expression in HCC cells. A) Proportion of upregulated and downregulated DEGs identified in RNA‐seq. B) ChIP‐seq signal coverages of histone/histone markers around the TSS of downregulated genes. C) Nucleosome‐free and mono‐nucleosome signals identified from ATAC‐seq around the TSS of downregulated genes. D) Western blot of total amount of H2A.Zac, H3K27ac, H3K4me1 and H2A.Z in VPS72 overexpressing Huh7 cells. E) Western blot of total amount of H2A.Zac and H2A.Z in VPS72 knockdown PLC/PRF/5 cells. F) RT‐PCR validation of genes downregulation indicated by RNA‐Seq, *n* = 3 independent experiments for (f).

To comprehensively investigate the regulatory role of VPS72, we performed RNA‐seq, ChIP‐seq, and ATAC‐seq in Huh7 cells overexpressing VPS72. We identified 182 differentially expressed genes (DEGs) with over 1.5‐fold changes in the RNA‐seq analysis upon VPS72 overexpression. Among these DEGs, only 40 genes were upregulated, while 142 genes were downregulated. Further filtering by a p‐value < 0.01 and a fold change >1.5 revealed that 13 genes were upregulated and 90 genes were downregulated (**Figure** **2**A). Among the top 30 DEGs listed in Table S6 (Supporting Information), 15 genes with moderate to high expression (log2 transformed Average Expression >2) were selected for qRT‐PCR validation. Downregulation of 11 genes was confirmed, supporting that VPS72 overexpression mainly represses gene expression in HCC cells (Figure 2F).

The widespread repression observed in the RNA‐seq data was unexpected. To explore this further, we examined ChIP‐seq and ATAC‐seq signals around the transcription start sites (TSS) of the downregulated genes (Figure 2B,C). As the chaperone necessary for the deposition of H2A.Z to chromatin to replace the canonical H2A in nucleosomes, VPS72 is known to directly interact with H2A.Z.^[^ ^14^ ^]^ In particular, H2A.Z acetylation (H2A.Zac) is enriched at the promoter of genes in active transcription.^[^ ^19^ ^]^ Surprisingly, the ChIP‐seq results showed that the H2A.Z signal decreased at the center and downstream of the TSS of repressed genes upon VPS72 overexpression. Additionally, the ChIP‐seq signals from the activation markers—H2A.Zac and H3K27ac—around the TSS were significantly less than those from the control group, indicating a repression pattern at these gene promoters (Figure 2B). Remarkably, there was an upregulation of the H3K4me1 signals surrounding the TSS sites. According to recent research, H3K4me1 at TSS was positively associated with DNA methylation when the methylation level is in the intermediate range, different from other active markers.^[^ ^20^ ^]^ H3K4me1 pre‐marks certain promoter CpG islands before aberrant DNA methylation, which is associated with gene repression.^[^ ^21^ ^]^ Importantly, VPS72 overexpression decreased H2A.Z acetylation levels, as confirmed by western blot analysis (Figure 2D). In contrast, VPS72 knockdown resulted in elevated H2A.Zac levels in HCC cells (Figure 2E). The H2A.Zac antibody we used recognizes acetylation at K4, K7, and K11 sites, which are known to be associated with active chromatin at promoter regions, promoting nucleosome destabilization and an open chromatin conformation.^[^ ^22^ ^]^ Excessive VPS72 may sequester H2A.Z from the enzyme acetyltransferase Tip60, which is responsible for acetylating H2A.Z.^[^ ^23^, ^24^ ^]^

The ATAC‐seq analysis, which reflects chromatin accessibility, showed no significant changes in nucleosome‐free and mono‐nucleosome signals at the repressed gene TSS regions (Figure 2C). This suggests that VPS72 might not broadly reshape chromatin structure at these TSS regions or that the regulation pattern is gene‐specific and not captured by stacked signals. In summary, VPS72 overexpression inhibited gene expression due to the decreased H2A.Zac level and repressive chromatin pattern surrounding the target genes’ TSS in HCC cells.

### 2.3 VPS72 Activates Lipogenesis in HCC Cells

**Figure 3:**
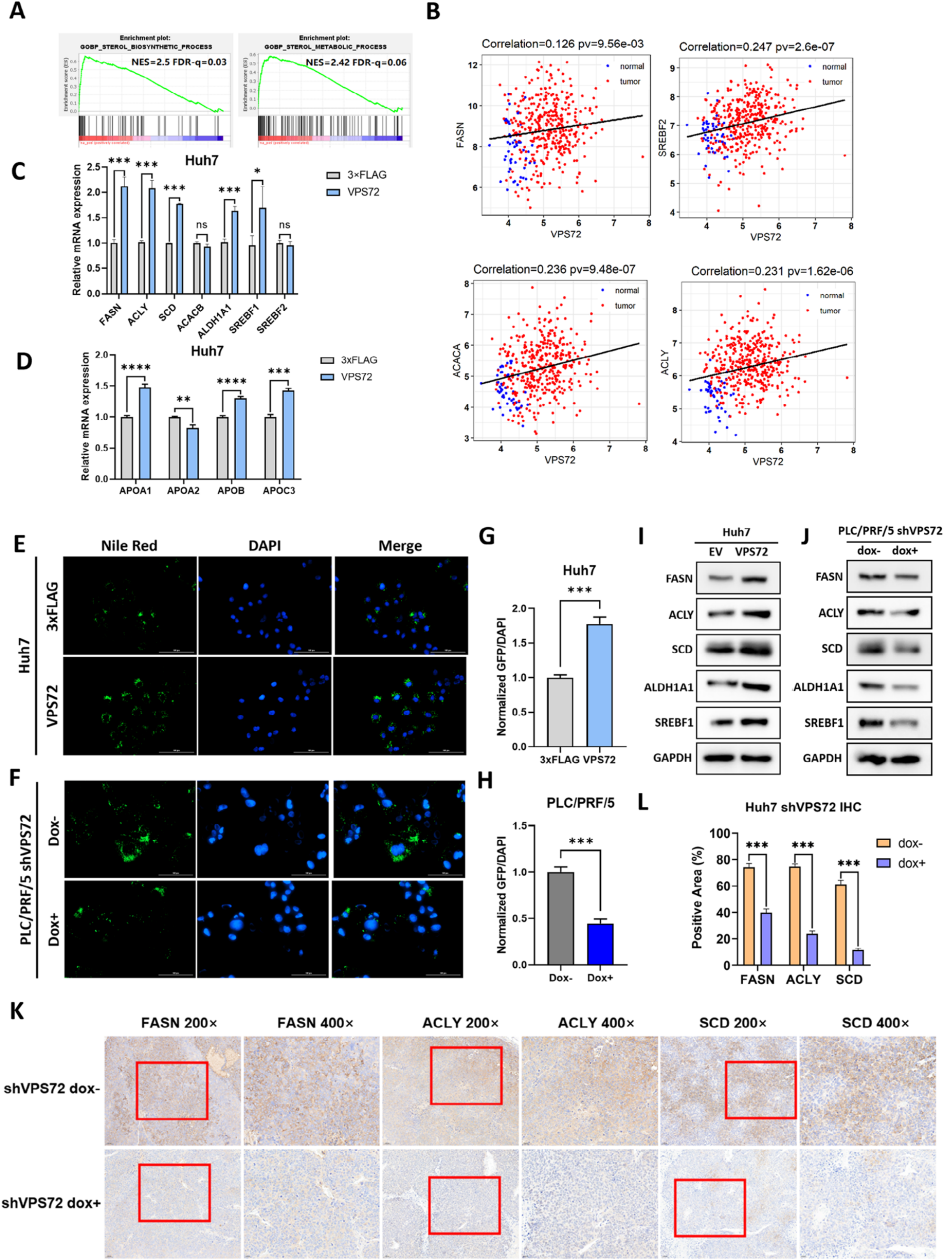
RNA‐seq analysis revealed activation of lipogenesis upon overexpression of VPS72. A) GSEA (Gene Set Enrichment Analysis) of the biological process of the RNA‐seq results. B) Correlation between VPS72 and key lipogenic genes with p‐value (pv) based on the TCGA LIHC RNA‐seq datasets. C) RT‐PCR validation of key lipogenic genes and D) lipoprotein‐associated genes upon VPS72 overexpression. E) Fluorescence pictures of VPS72 overexpressing Huh7 cells and F) VPS72 knockdown PLC/PRF/5 cells. Nile red fluorescence was captured under GFP channel, and Hoechst fluorescence was captured under DAPI channel. G) Statistics of lipid droplets from Huh7 cells upon VPS72 overexpression and H) PLC/PRF/5 cells upon VPS72 knockdown, with the signal intensity from GFP channel was normalized by DAPI signal intensity. I) Western blot of FASN, ACLY, SCD, ALDH1A1, and SREBF1 in VPS72 overexpressing Huh7 cells and J) VPS72 knockdown PLC/PRF/5 cells. K) IHC of tumor tissue of nude mice used in the mouse xenograft model. L) Quantification of the positive area of IHC results. *n* = 3 independent experiments for (c) and (d), *n* = 3 subjects per group in (f) and (g); *n* = 5 subjects per group in (k).

In order to investigate the function of VPS72 in HCC, gene set enrichment analysis (GSEA) of RNA‐seq data was performed on all genes after they were ranked. GSEA indicated a significant enrichment of biological processes related to sterol/cholesterol biosynthesis and homeostasis, as well as lipoprotein‐associated processes upon VPS72 overexpression (**Figure** **3**A; Figure S4, Supporting Information). Additionally, hallmarks such as E2F targets, mTORC1 signaling, and MYC targets were prominently enriched upon VPS72 overexpression (Figure S4, Supporting Information).

To validate the impact of VPS72 on lipogenesis, we performed a correlation study between VPS72 expression and important lipogenic genes (e.g., FASN, ACLY, SREBF2) and mTORC1 signaling using the TCGA LIHC dataset. The analysis revealed a positive correlation between VPS72 expression and the expression of these genes/pathway, supporting its role in regulating the lipogenesis biological process (Figure 3B). A positive correlation between VPS72 expression and key lipogenic or lipoprotein‐associated genes (SREBF1, SREBF2, FASN, ACLY, SCD, ALDH1A1, APOA1, APOB, and APOC3) can be also identified from our RNA‐seq data (Figure S5A, Supporting Information).

Among these genes, SREBF1 and SREBF2 are key transcription factors controlling lipid biosynthesis, activating the expression of FASN, ACLY, and SCD, which are crucial enzymes in the lipid biosynthesis process.^[^ ^25^, ^26^ ^]^ Although ALDH1A1 (Aldehyde Dehydrogenase 1 Family Member A1) is not a core participant in lipogenesis, its transcription is reported to be activated by SREBF1.^[^ ^27^ ^]^ The activation of ALDH1A1 expression further supports the transcriptional activation of SREBF1 target genes. To confirm these findings, we examined the expression levels of key lipogenic genes using RT‐PCR in Huh7 cells overexpressing VPS72. The results showed consistent upregulation trends, aligning with RNA‐seq and correlation analysis data (Figure 3C).

The expression of the key lipogenic enzymes is further confirmed by IHC and Western blot experiments. Western blot analysis demonstrated that VPS72 overexpression markedly upregulated the protein levels of FASN, ACLY, SCD, ALDH1A1, and SREBF1, whereas VPS72 knockdown led to their downregulation in HCC cells (Figure 3I,J). Consistently, IHC assays confirmed decreased expression of FASN, ACLY, and SCD in tumor tissues derived from VPS72 knockdown cells (Figure 3K,L). These findings align with mRNA‐level data, underscoring the role of VPS72 in enhancing lipogenesis.

Given the importance of these activated lipogenic genes, it is reasonable to presume that lipid homeostasis in HCC cells is affected by aberrant VPS72 expression. Activation of these key enzymes should accelerate the production of lipid droplets and the secretion of lipoproteins since lipid biosynthesis is a primary function of hepatic cells. The activated expression of apolipoproteins (APOA1, APOB, APOC3) under high VPS72 levels suggests that increased lipid biosynthesis may require more apolipoproteins for lipoprotein secretion (Figure 3D).

Lipid droplets stored within HCC cells serve multiple purposes, including providing energy and generating acetyl‐CoA for cell signaling. Therefore, the accumulation of lipid droplets in HCC is considered an important phenotypic metric. To validate lipid droplet accumulation, Huh7 and PLC/PRF/5 cells were fixed and stained with Nile Red, allowing visualization of lipid droplets in the cytosol under a fluorescent microscope (Figure 3E–H). As expected, VPS72 overexpression led to lipid droplet accumulation in Huh7 cells, while VPS72 knockdown in PLC/PRF/5 cells resulted in fewer lipid droplets per cell.

### 2.4 VPS72 Promotes Lipogenesis by Activating mTORC1 Signaling

Next, we aimed to investigate the upstream pathway involved in lipogenesis mediated by VPS72. GSEA analysis of our RNA‐seq data revealed significant activation of the mTORC1 signaling pathway upon VPS72 overexpression (Figure S4, Supporting Information). The mTORC1 pathway plays a crucial role in regulating cell metabolism, proliferation, and survival.^[^ ^28^ ^]^ Notably, mTORC1 has been reported to activate the expression of lipogenic enzymes through the regulation of SREBF1.^[^ ^11^, ^29^, ^30^ ^]^

**Figure 4:**
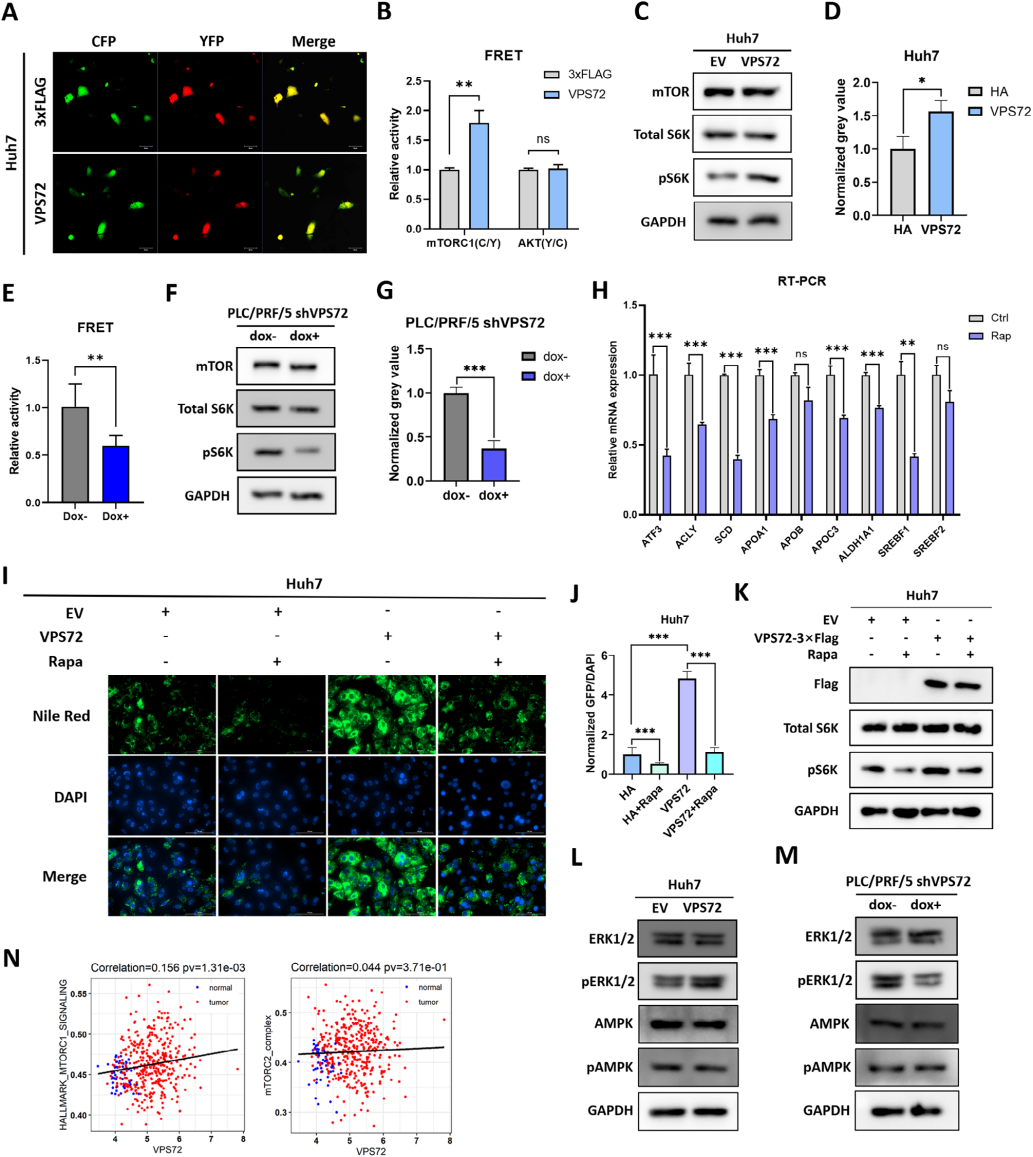
VPS72 activated mTORC1 signaling and promoted lipogenesis in HCC cells. A) FRET reporter images of mTORC1 activity. B) Statistics of mTORC1 and Akt reporter activities in Huh7 cells overexpressing VPS72. C) Western blot of the phosphorylation level of mTORC1 substrate S6K in Huh7 cells. D) Quantification of the grey value of (c). E) Statistics of mTORC1 reporter activity in PLC/PRF/5 cells with VPS72 knockdown. F) Western blot of the phosphorylation level of mTORC1 substrate S6K in PLC/PRF/5 cells with VPS72 knockdown. G) Quantification of the grey value of (f). H) RT‐PCR results of the expression of key lipogenesis enzymes and apolipoproteins after 24 h 100 nM rapamycin treatment. I) Images and J) Statistics of Nile red staining of Huh7 cells overexpressing VPS72 or empty vector (EV) with or without 100 nM rapamycin treatment for 24h. K) Western blot of the phosphorylation level of mTORC1 substrate S6K in Huh7 cells overexpressing VPS72 or EV with or without 100 nM rapamycin treatment for 24h. L) Western blot of AMPK, phosphorylated AMPK, ERK1/2, phosphorylated ERK1/2 in VPS72 overexpressing Huh7 cells and M) VPS72 knockdown PLC/PRF/5 cells. N) Correlation between VPS72 and mTORC1 signaling hallmark and mTORC2 complex components based on TCGA LIHC RNA‐Seq datasets. *n* = 3 subjects per group in (b), (e), and (j); *n* = 3 independent experiments for (d), (g) and (h).

To validate mTORC1 activity, we employed a fluorescence resonance energy transfer (FRET)‐based kinase activity biosensor reporter system, TORCAR.^[^ ^31^ ^]^ Western blotting was also performed to assess the phosphorylation level of the mTORC1 substrate S6K. Both FRET and western blot results indicated elevated mTORC1 complex activity upon VPS72 overexpression in Huh7 cells (**Figure** **4**A–D). Conversely, repression of mTORC1 activity was observed in PLC/PRF/5 cells with VPS72 knockdown (Figure 4E–G). Interestingly, the Akt reporter showed no significant difference upon treatment (Figure 4B). Given that Akt is a substrate of the mTORC2 complex, this suggests that VPS72 may not significantly influence mTORC2 activity, which has also been reported to regulate lipid metabolism in HCC.^[^ ^6^, ^32^ ^]^

To further investigate the central role of mTORC1 activation in VPS72‐mediated lipid accumulation, we treated cells with rapamycin to inhibit mTORC1 activity. Rapamycin is a specific inhibitor that acutely and directly inhibits mTORC1, but only chronic administration affects mTORC2 activity.^[^ ^33^ ^]^ Cells treated with 100 nM rapamycin for 24 h were examined for the expression of lipogenesis and apolipoprotein genes. All VPS72‐activated genes were suppressed by rapamycin treatment, including the key regulator SREBF1 (Figure 4H).

Lipid droplets were visualized using Nile Red staining. A significant decrease in lipid droplets per cell was observed in the rapamycin‐treated group (Figure 4I,J). Furthermore, rapamycin treatment effectively reversed the enhanced lipid accumulation caused by VPS72 overexpression (Figure 4I,J). Western blot analysis further confirmed that VPS72 overexpression activated mTORC1 signaling, as indicated by increased phosphorylation of S6K. Notably, this activation was abrogated upon Rapamycin treatment, underscoring the critical role of mTORC1 in VPS72‐induced lipogenesis (Figure 4K). These results indicate that the mTORC1 signaling pathway plays a central role in regulating lipid synthesis and accumulation in HCC cells. The activation of mTORC1 in VPS72‐overexpressing cells is a key factor contributing to upregulated lipid biosynthesis and accumulation.

Given that AKT is not implicated in VPS72‐mediated mTORC1 activation, we evaluated the activity of two other upstream kinases, AMPK and ERK1/2, in VPS72‐overexpression (VPS72‐OE) and VPS72‐knockdown (VPS72‐KD) HCC cell lines. Our analysis revealed that only ERK1/2 activity was significantly elevated in VPS72‐OE cells, whereas its activity was reduced in VPS72‐KD cells (Figure 4L,M). These findings suggest that mTORC1 activity is regulated by VPS72 through ERK1/2 signaling pathways. To evaluate the impact of ERK1/2 suppression on HCC cell growth, we treated HCC cell lines with the well‐established MEK/ERK1/2 inhibitor mirdametinib^[^ ^34^ ^]^ and conducted western blot and cell proliferation assays. The results demonstrated that mirdametinib significantly inhibited HCC cell proliferation, suggesting that ERK1/2 could serve as a potential therapeutic target for HCC treatment (Figure S6, Supporting Information).

Furthermore, we examined the correlation between VPS72 expression and key components of mTOR signaling pathways, including the hallmark of mTORC1 signaling and elements of the mTORC2 complex, based on TCGA LIHC dataset. VPS72 expression and the mTORC1 signaling signature were shown to be significantly positively correlated, with minor correlations seen between VPS72 expression and mTORC2 complex components (Figure 4N). A similar correlation can be observed from our RNA‐seq data (Figure S5B, Supporting Information).

### 2.5 VPS72 Represses ATF3 Expression to Activate mTORC1 Signaling

**Figure 5:**
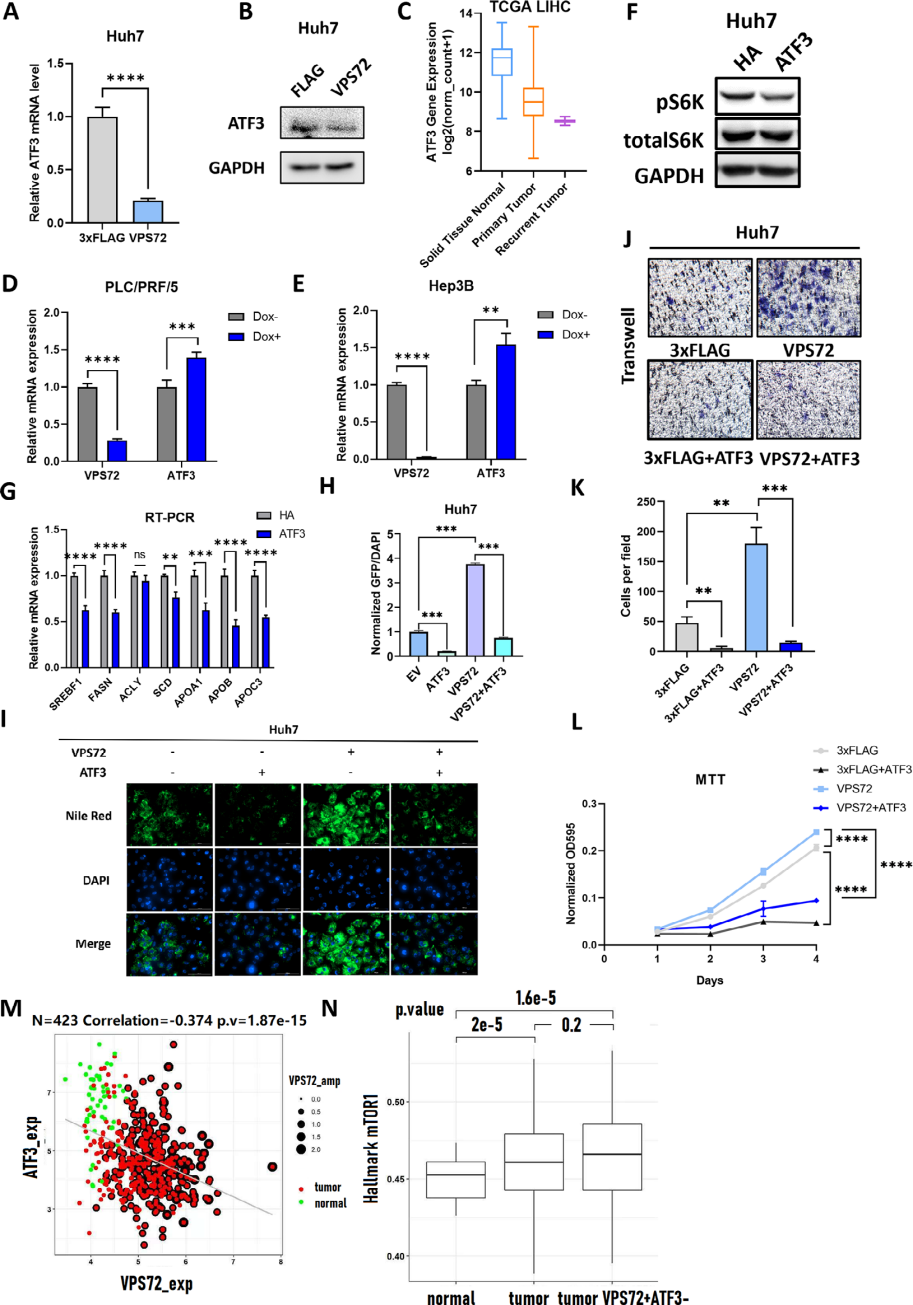
VPS72 repressed ATF3 expression with elevated mTORC1 activity and lipogenesis. A) RT‐PCR and B) Western Blot result of ATF3 level upon overexpression of VPS72 in Huh7 cells. C) ATF3 expression level comparison, tumor versus normal tissue, based on TCGA LIHC datasets. D) RT‐PCR result of ATF3 expression in PLC/PRF/5 and (E) Hep3B cells upon VPS72 knockdown. F) Western blot of phosphorylated S6K level, and G) RT‐PCR of lipid metabolism gene expression level upon ATF3 overexpression. H) Statistics and I) images of Nile Red staining of lipid droplets from Huh7 cells overexpressing ATF3, VPS72, or both. J) Transwell assay of Huh7 cells overexpressing ATF3, VPS72, or both with K) statistics. L) MTT assay of Huh7 cells overexpressing ATF3, VPS72, or both. M) Correlation analysis between VPS72 and ATF3 expression in TCGA LIHC patients. N) mTORC1 activity from TCGA LIHC samples, stratified into three groups, normal, tumor, and tumors with VPS72 copy number gain (>median expression) and ATF3 loss (<median expression). *n* = 3 independent experiments for (a), (d), (e), (g), (h), (k), and (l).

ATF3 is known to repress mTOR activity in hepatic cells and was one of the top DEGs identified in our RNA‐seq results (Figure 2A). It is highly likely that ATF3 links VPS72 to mTORC1 regulation. We first validated the regulatory effect of VPS72 on ATF3 expression. In Huh7 cells, ATF3 levels were significantly repressed by VPS72, as demonstrated by both RT‐PCR and western blot (**Figure** **5**A,B). Notably, ATF3 mRNA levels in TCGA LIHC patients are significantly lower than in normal tissues, indicating recurrent repression of ATF3 expression in HCC (Figure 5C). Furthermore, the knockdown of VPS72 in PLC/PRF/5 and Hep3B cells resulted in activated ATF3 expression, as shown by RT‐PCR (Figure 5D–E). These results collectively indicate that VPS72 negatively regulates ATF3 expression in HCC cells.

To validate the role of ATF3 in VPS72‐mediated activation of mTORC1 signaling, we examined the phosphorylation level of the mTORC1 substrate S6K by western blot. The phosphorylated S6K level decreased upon ATF3 overexpression in Huh7 cells (Figure 5F), indicating that mTORC1 complex activity was repressed by ATF3. Consistent with this, key lipid metabolism genes downstream of mTORC1 were downregulated under ATF3 overexpression, as demonstrated by RT‐PCR (Figure 5G). Consequently, a reduced amount of lipid droplets was observed in ATF3‐overexpressing cells. Additionally, ATF3 overexpression reversed the accumulated lipid droplet phenotype caused by VPS72 overexpression (Figure 5H,I).

Overexpression of ATF3 in VPS72‐overexpressing Huh7 cells reversed the enhanced migration and proliferation observed in these cells (Figure 5J–L). Western blot analysis confirmed the successful overexpression of both VPS72 and ATF3. Importantly, ATF3 overexpression counteracted VPS72‐induced ERK1/2 activation, suggesting that VPS72 activates ERK1/2 by suppressing ATF3 expression (Figure S7, Supporting Information). Moreover, ATF3 overexpression significantly reduced cell growth, as demonstrated by the MTT assay, underscoring its potent inhibitory effect on HCC cell proliferation (Figure 5L).

To further elucidate the relationship between VPS72 and ATF3 expression, as well as their connection to mTORC1 activity in liver cancer, we conducted data mining of liver cancer expression datasets. Analysis of the TCGA LIHC database revealed a negative correlation between VPS72 and ATF3 expression, indicating that VPS72 amplification may downregulate ATF3 in liver cancer (Figure 5M). Further investigation of the TCGA LIHC data showed that mTORC1 signaling is generally activated in tumors compared to normal tissues. Notably, tumors with VPS72 copy number gain (>median expression) and ATF3 loss (<median expression) exhibited heightened mTORC1 activation, suggesting that mTORC1 signaling is a downstream target of VPS72 amplification in liver cancer (Figure 5N). These findings suggest that ATF3 regulates mTORC1 activity, which in turn modulates lipid metabolism and contributes to HCC progression.

### 2.6 VPS72 Overexpression Epigenetically Modulates ATF3 Promoter and Represses its Expression

**Figure 6:**
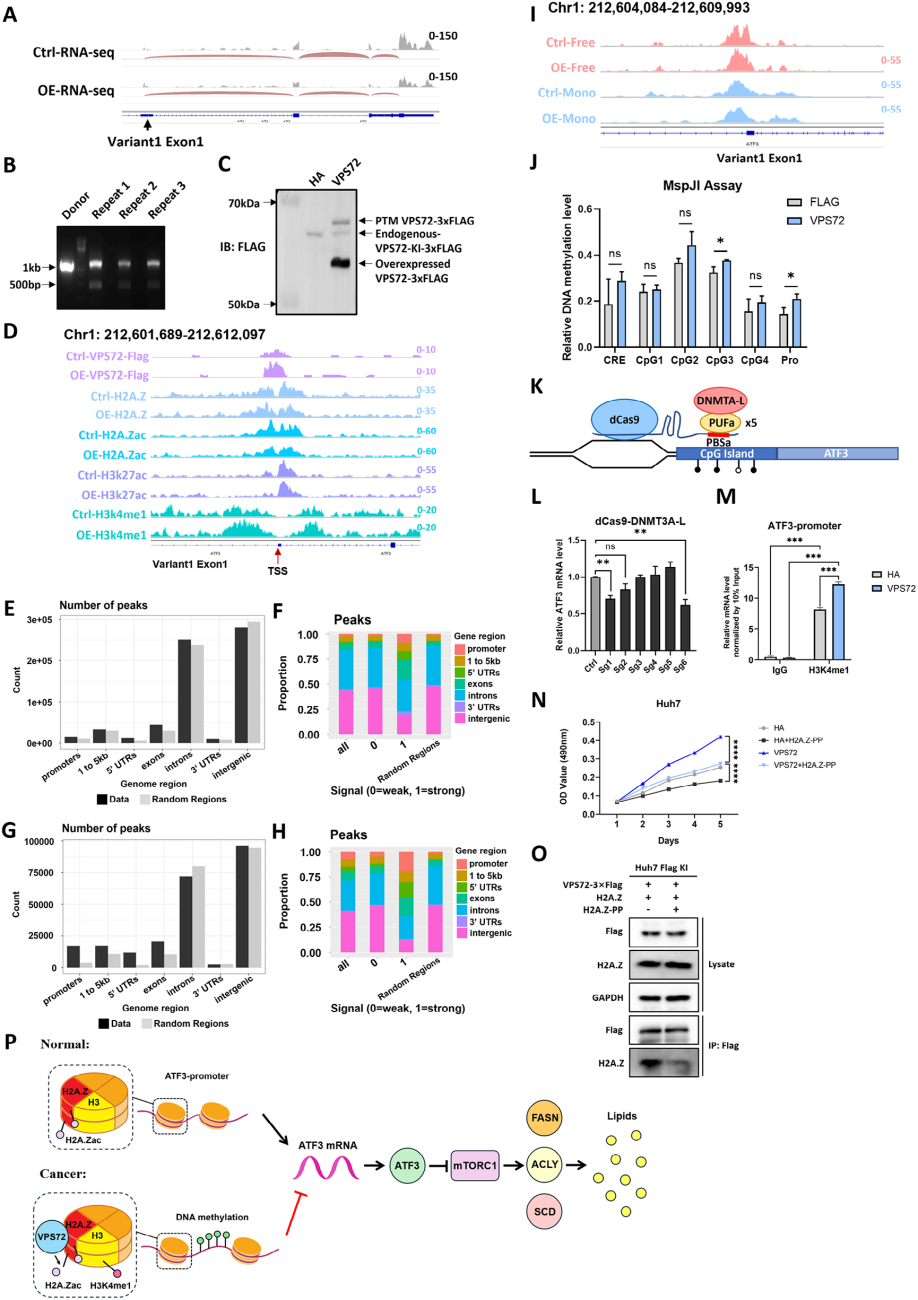
VPS72 overexpression epigenetically modulates ATF3 promoter and represses its expression. A) RNAs‐seq coverage information identified ATF3 variant1 is the dominant variant expressed in Huh7 cells. B) Genotyping PCR result, positive band 1100 bp, negative band 473bp. C) Western blotting result, endogenous VPS72 with FLAG tags showed a ≈63 kDa band, viral expressed VPS72‐3xFLAG showed two bands at ≈58 and ≈67kDa. D) ChIP‐seq signals around the TSS of ATF3 variant 1. E) ChIP‐seq peak distribution of count and F) proportion of VPS72‐FLAG signals, and the G) count and H) proportion of H2A.Z signals. I) ATAC‐seq signals around the TSS of ATF3 variant 1. Free: Nucleosome‐free signals; Mono: Mono‐nucleosome signals. J) Methylation levels around and at the ATF3 promoter. Methylation levels were normalized to the control group with no MspJI enzyme digestion. CRE: cis‐regulatory element, Pro: promoter. (K) Schematic mechanism of dCas9‐DNMT directed DNA methylation assay. L) RT‐PCR results of DNA methylation assay. M) ChIP‐ qPCR assay of H3K4me1 on the promoter region of ATF3. N) MTT assay of Huh7 cells with overexpression of empty lenti‐HA vector or VPS72 alone or combined with H2A.Z peptide. O) Co‐IP assay of Huh7 FLAG‐KI cells with overexpression of empty lenti‐HA vector or lenti‐HA vector expressing H2A.Z peptide. P) Schematic figure of the mechanism of VPS72‐induced ATF3 repression and lipogenesis inhibition. *n* = 3 independent experiments for (j), (l), (m), and (n).

ATF3 variants are reported to be transcribed from two promoters, with the two respective full‐length variants (variant 1 and variant 3) encoding the same protein isoform.^[^ ^35^ ^]^ From our RNA‐seq results, we identified that Huh7 cells express variant 1 of ATF3 (**Figure** **6**A). Consequently, ChIP‐seq and ATAC‐seq signals surrounding the promoter for variant 1 were studied to investigate the repression of the ATF3 promoter.

To study VPS72 more precisely at the endogenous level, we utilized a CRISPR‐Cas9 system‐based knock‐in (KI) technique to add FLAG tags at the end of the endogenous VPS72 protein coding sequence. Following the transfection of the CRISPR‐Cas9 components and template plasmids, the genome‐edited cells underwent drug selection, and the surviving colonies (referred to as Huh7‐KI cells) were subjected to genotyping. PCR analysis confirmed the successful integration of the exogenous sequences (Figure 6B). Traces of negative bands were observed, which could indicate possible contamination with genome‐unmodified cells. However, this should not affect subsequent experiments.

Western blot analysis successfully detected the endogenous VPS72‐KI‐3xFLAG fusion protein using FLAG antibodies (Figure 6C). It's important to note that the VPS72‐3xFLAG fusion protein overexpressed by the lentiviral system carries a 3xFLAG tag, which differs from the three tandem FLAG tags in the endogenous KI version, resulting in a smaller molecular weight as shown in the western blot results. Interestingly, upon VPS72 overexpression, a distinct band with a higher molecular weight was detected. This band may represent a post‐translationally modified (PTM) form of VPS72, as previous studies have reported phosphorylation of VPS72 at amino acids 127 and 129.^[^ ^36^, ^37^ ^]^

With the successful construction of the knock‐in cell line, we proceeded with ChIP‐Seq assays to further investigate the role of VPS72 in chromatin regulation and gene expression. As a chaperone of H2A.Z, VPS72 can directly regulate ATF3 expression by modulating H2A.Zac and H3K4me1 levels at the ATF3 promoter region. ChIP‐seq results revealed enriched VPS72‐Flag signals flanking the transcription start site (TSS) of ATF3 (signals in the region of chr1: 212605485‐ 212607140, −1503 to + 152 of ATF3 promoter). In control cells, H2A.Z signals were more abundant downstream of the TSS. However, in VPS72‐overexpressing cells, H2A.Z signal pattern around the TSS was changed compared to the control group. Notably, H2A.Zac signals—associated with active transcription—were markedly reduced in VPS72‐overexpressing cells, while H3K4me1 signals were strikingly enhanced. In contrast, there were no significant changes in H3K27ac signals (Figure 6D).

The statistical evaluation of the percentage and number of ChIP‐Seq peaks for VPS72‐Flag and H2A.Z revealed similar patterns (Figure 6E–H). The similarity in peak distribution suggests that VPS72 may facilitate the incorporation or stabilization of H2A.Z at specific genomic loci, contributing to chromatin remodeling and gene regulation. The overlap in their ChIP‐Seq profiles underscores the close relationship between these two proteins in modulating chromatin structure and function in HCC cells.

We further examined the ATAC‐seq signals at the ATF3 promoter region, which can be used to identify nucleosome‐free and mono‐nucleosome areas around the TSS sites. Interestingly, control cells showed a bimodal distribution pattern in nucleosome‐free regions around the TSS sites, representing the active binding of transcription factors or RNA polymerase II involved in the transcription of ATF3 (signal in the region of chr1: 212606580–212607698, −408 to +710 of the promoter). However, VPS72 overexpression cells showed a monomodal distribution pattern for nucleosome‐free regions around the TSS sites with only the upstream peak remaining detectable (Figure 6I). These results imply that overexpression of VPS72 alters chromatin accessibility.

Interestingly, the H3K4me1 signals were significantly upregulated upon VPS72 overexpression (signal in the region of chr1: 212605229–212607334, −1759 to +346 of the promoter) (Figure 6D). Recent studies have shown that, in contrast to other active markers, H3K4me1 at TSS was positively correlated with DNA methylation when the methylation level was in the intermediate range.^[^ ^20^ ^]^ Gene repression and abnormal DNA methylation were linked to H3K4me1 marks at the promoter CpG islands.^[^ ^21^ ^]^ This prompts us to investigate the methylation level proximal to the TSS site of ATF3 using a methylation‐sensitive restriction enzyme MspJI‐based assay. Genomic DNA was extracted from cells and subjected to MspJI enzyme digestion, which selectively digests methylated DNA. RT‐PCR was then used to quantify the remaining unmethylated DNA. The results indicate that the relative DNA methylation levels around the ATF3 TSS were slightly elevated upon overexpression of VPS72 (Figure 6J).

To demonstrate that ATF3 expression is linked to its promoter methylation level, we modified the DNA methylation level at the CpG island area using a dCas9‐DNMT3A‐L system (Figure 6K). The results showed that the expression level of ATF3 was significantly suppressed by sgRNA1 and sgRNA6 (Figure 6L). In addition, ChIP‐qPCR analysis confirmed the upregulation of H3K4me1 at the ATF3 promoter, consistent with our ChIP‐seq findings, further elucidating the epigenetic regulation of ATF3 by VPS72 (Figure 6M).

To explore the therapeutic potential of disrupting the interaction between VPS72 and H2A.Z, we designed a peptide (H2A.Z‐PP) based on the previously characterized interaction region of H2A.Z with VPS72.^[^ ^15^ ^]^ The peptide‐encoding DNA was cloned into a lenti‐HA vector for experimental evaluation. MTT assays demonstrated that while VPS72 enhances HCC cell proliferation, H2A.Z‐PP effectively counteracted this effect, underscoring its potential to suppress tumor growth (Figure 6N). Furthermore, co‐immunoprecipitation (co‐IP) assays confirmed that H2A.Z‐PP effectively disrupted the interaction between VPS72 and H2A.Z (Figure 6O). These findings highlight the VPS72‐H2A.Z interaction as a critical axis in HCC progression and position H2A.Z‐PP as a promising therapeutic agent for targeting this pathway.

In summary, VPS72 represses ATF3 expression through multiple mechanisms, including chromatin accessibility alterations with reduced H2A.Z acetylation and incorporation along with the increase of DNA methylation near the TSS. The reduction of H2A.Z acetylation level and increase of DNA methylation around the TSS of ATF3 play a dominant role in repressing ATF3 transcription. These findings provide new insights into the epigenetic regulation of ATF3 by VPS72 and its implications for HCC progression and therapeutic targeting (Figure 6P).

## 3 Discussion

Metabolic reprogramming is a hallmark of cancer, enabling tumor cells to adapt to their microenvironment and sustain rapid proliferation. While the Warburg effect and glutaminolysis have long been recognized as key metabolic adaptations,^[^ ^38^ ^]^ lipid metabolism has emerged as a crucial driver of tumor progression.^[^ ^39^, ^40^, ^41^ ^]^ Lipid metabolism reprogramming in hepatocellular carcinoma is often characterized by the upregulation of key lipogenic genes such as ACLY, ACC, FASN, and SCD, regulated by the SREBF1 pathway.^[^ ^42^, ^43^ ^]^ This shift enhances the synthesis of fatty acids and cholesterol, which are indispensable for tumor growth, survival, and metastasis.

Moreover, accumulating evidence links lipid metabolism to epigenetic regulation.^[^ ^44^ ^]^ Fatty acid intermediates such as acetyl‐CoA and succinyl‐CoA serve not only as precursors for lipid synthesis but also as cofactors for histone modifications, including acetylation and succinylation. This dual role establishes a feedback loop where lipid metabolism drives epigenetic changes that reinforce metabolic reprogramming. Such dynamic crosstalk highlights the profound interplay between lipid metabolism and gene regulation in cancer.

While the oncogenic role of H2A.Z—a histone variant critical for chromatin dynamics—has been increasingly recognized in cancer, its chaperone VPS72 has remained largely understudied. In this investigation, we demonstrate the significance of VPS72 as a driver of HCC progression through its regulation of chromatin structure and lipid metabolism. Expanding on our previous work, which implicated VPS72 in poor prognosis, we provide mechanistic insights into how VPS72 exerts its oncogenic effects by orchestrating the VPS72‐ATF3‐mTORC1 axis.

Our findings establish VPS72 as a critical regulator of lipid metabolism and tumorigenesis in HCC. Specifically, we demonstrate that VPS72 represses ATF3, a stress‐responsive master regulator of cellular metabolism and immunity.^[^ ^45^ ^]^ ATF3 suppression by VPS72 activates the mTORC1 signaling pathway, promoting lipogenesis and enhancing tumor growth and metastasis. These results are further supported by the study of TCGA LIHC patient data, which shows that VPS72 expression is negatively correlated with ATF3 expression but positively correlated with the activity of the mTORC1 signaling pathway and the expression of important lipogenic genes. This is especially noteworthy because ATF3 suppresses tumors in hepatocytes by reducing inflammatory damage and metabolic imbalance.^[^ ^46^ ^]^ A significant aspect of our study is the discovery that VPS72‐mediated repression of ATF3 occurs via epigenetic mechanisms, including the incorporation of H2A.Z at the ATF3 promoter, and modulation of H2A.Z acetylation state and the methylation level of the associated DNA. These findings provide a compelling link between chromatin remodeling and metabolic regulation in cancer.

Our integrative analyses using RNA‐seq, ChIP‐seq, and ATAC‐seq highlight the broad transcriptional repression associated with VPS72 overexpression. The uneven effects of VPS72 on gene expression, despite its lack of sequence specificity, underscore the complexity of its regulatory role. Unlike transcription factors that target specific DNA motifs, VPS72 likely exerts its effects through chromatin context‐dependent mechanisms, including histone variant deposition and epigenetic modifications. This raises intriguing questions about how VPS72 selectively influences gene expression, particularly its preference for regulating metabolic genes such as ATF3.

Intriguingly, VPS72 overexpression reduced H2A.Z acetylation levels, whereas VPS72 knockdown boosted them in HCC cells. It is plausible that excessive VPS72 might sequester H2A.Z from the acetyltransferase Tip60 enzyme, which is responsible for acetylating H2A.Z.^[^ ^23^, ^24^ ^]^ In addition to H2A.Zac signal reduction around the TSS sites, and the increase of H3K4me1 signals were observed at the same sites. Different from other active markers, H3K4me1 signals at TSS were positively associated with DNA methylation when the methylation level is in the intermediate range.^[^ ^20^ ^]^ It needs further investigation how the interplay between H2A.Zac, H3K4me1, and DNA methylation at TSS sites affect gene regulation and cancer development.

The VPS72‐ATF3‐mTORC1‐lipogenesis axis identified in this study not only underscores the oncogenic potential of VPS72 but also establishes a new paradigm for understanding the epigenetic regulation of metabolic reprogramming in HCC. Given the central role of ATF3 in regulating diverse metabolic pathways, including gluconeogenesis and adipocyte browning, aberrant VPS72 expression may have broader metabolic consequences beyond lipid metabolism.

Our findings also raise the possibility of therapeutic targeting of VPS72 or its interaction with H2A.Z. The disruption of this axis could offer a novel strategy to inhibit lipid biosynthesis and tumor growth. Future studies should aim to elucidate additional VPS72‐regulated pathways and explore its potential as a therapeutic target across different cancer types.

Notably, the copy number gains of VPS72 were found in over half of the TCGA LIHC samples, underscoring the prevalence of this mechanism in the disease. However, our analysis also revealed that VPS72 expression is comparatively lower in non‐alcoholic steatohepatitis (NASH)‐driven HCC compared to alcohol‐, HBV‐, or HCV‐driven cases. This suggests that NASH‐driven HCC may rely less on VPS72 amplification to initiate cancer development, indicating a potential divergence in the underlying oncogenic pathways.

In summary, this study establishes VPS72 as a pivotal oncogene in HCC by linking its chromatin remodeling activity to metabolic reprogramming via the ATF3‐mTORC1 axis. By repressing ATF3, VPS72 drives lipid biosynthesis and tumor progression, highlighting a novel mechanism of metabolic regulation in cancer. The dual role of VPS72 in chromatin remodeling and metabolic control underscores its significance as a therapeutic target, offering new avenues for combating HCC and potentially other malignancies.

## 4 Experimental Section

### Cell Maintenance

Human HCC cell lines PLC/PRF/5, Hep3B, Huh7, and LO2 were purchased from the Japanese Collection of Research Bioresources Cell Bank ((JCRB, Osaka, Japan). The human kidney cell line Lenti‐X 293T was purchased from Takara. All cells were cultured in Dulbecco's modified Eagle medium (DMEM, Gibco). 10% fetal bovine serum (FBS, Gibco) and antibiotic‐antimycotic mixture (Gibco) were added into the culture media. Cells were maintained at 37 °C in a CO2 incubator with 5% CO2. Doxycycline was added to culture media at a final concentration of 1 µg ml^−1^ to induce the expression of VPS72 shRNA.

### Molecular Cloning

The coding sequence (CDS) of VPS72 was amplified from Huh7 cDNA, and the CDS of ATF3 was cloned from pRK‐ATF3 (26 115, Addgene). To express VPS72 or ATF3 protein, lentiCas9‐Blast (52 962, Addgene) was used to create a lenti‐viral vector (lenti‐3 × Flag) with two Esp3I cleavage sites and a 3xFlag tag. The CDS of VPS72 or ATF3 were cloned into the lenti‐3xFlag vector between the two Esp3I sites by Hot‐fusion plasmid assembly, as described before.^[^ ^47^ ^]^ VPS72 shRNA oligos were purchased from Integrated DNA Technologies (IDT, Hong Kong). All of the oligonucleotides were annealed and cloned into the previously described vector.^[^ ^48^ ^]^ The empty vector, lenti‐HA vector, is abbreviated as EV or HA. The primers sequences utilized in cloning were recorded in Table S1 (Supporting Information).

### Transfection and Lentivirus Production

Plasmids were transfected into cells by ViaFect (Promega). For lentivirus packaging, psPAX2, pMD2.G together with the carrier plasmid were co‐transfected into HEK293T cells in a 2:1:2 mass ratio. Lentivirus was harvested 48 and 72h after transfection by centrifuging at 500g for 5 min and filtering through a 0.45 um filter and preserved at −80 °C. Lentivirus concentration was achieved using PEG‐it Virus Precipitation Solution (SBI, Mountain View, CA).

### RNA Isolation and Quantitative Reverse Transcription PCR (RT‐qPCR)

Total RNA was isolated using RNAzol (Molecular Research Center). Reverse transcription was performed by following the manufacturer's instructions (K1622, Thermo Fisher Scientific). Quantitative Reverse Transcription PCR (RT‐qPCR) was conducted by using SYBR (Bio‐Rad) with the StepOnePlus Real‐Time PCR System (Applied Biosystems). Relative gene expression values were calculated by the standard ΔΔCt method. RT‐qPCR primer sequences were recorded in Table S2 (Supporting Information).

### Transwell Assay

The assay was conducted as previously described.^[^ ^48^ ^]^ Briefly, complete DMEM was added into 24‐well plates, and transwell inserts were placed into the wells. 1 × 10^5^ cells were then trypsinized, counted, and resuspended in DMEM without FBS and supplied to the inserts. The inserts were maintained in the incubator for 24 h and then collected, and the cells on the upside were wiped off. Cells were treated with 4% paraformaldehyde (PFA) for fixation and then stained using crystal violet, photographed under the microscope and counted manually.

### Wound Healing Assay

The assay was conducted in accordance with the previously outlined procedure.^[^ ^48^ ^]^ Briefly, Cells were seeded into a 6‐well plate and allowed to grow until the cell density exceeded 70%. A wound was manually created using a pipette tip. The culture media was then replaced with DMEM containing 1 mM thymidine and 2% FBS. Images of the wound area were captured under a microscope at 0 h and 24 h, and the area of the wound was measured using ImageJ software.

### Colony Formation Assay

The assay was conducted as previously described.^[^ ^48^ ^]^ Briefly, 2000 cells were seeded into a 6‐well plate. Cells were maintained, and culture media were changed every 3 days. Once the colonies exceeded 50 cells, they were fixed with 4% PFA, stained with crystal violet, photographed under the microscope, and counted manually.

### Cell Proliferation Assay

MTT assay was conducted as previously described.^[^ ^48^ ^]^ Briefly, 4000 cells were seeded into 96‐well plates.10µl 5mg ml^−1^ MTT was supplied into cells and kept at 37 °C for 3h. The media was then discarded, and 100µl DMSO was added into the wells. The plate was then kept on a shaker in the dark for 15 min. The absorbance was recorded at a wavelength of 595 nm. For the ERK1/2 inhibition assay, 2500 cells were seeded into 96‐well plates and treated with 20 µM mirdametinib at varying final concentrations. Cells were cultured for 72 h, with images captured every 12 h using the PAULA Cell Imager (Leica Microsystems). Cell numbers were quantified using ImageJ software.

### Western Blotting

The assay was conducted as previously described.^[^ ^48^ ^]^ Briefly, cells were lysed on ice with RIPA buffer supplied with protease and phosphatase inhibitors. The lysate was centrifuged at 13000 rpm for 5 min. The supernatant was collected and supplied with LDS Sample Buffer (Thermo Fisher Scientific) and heated at 100 °C for 15 min. The samples were then analyzed using SDS‐PAGE, followed by transferring the proteins onto PVDF membranes. The membranes were incubated in succession with 5% bovine serum albumin (BSA), primary antibodies, and secondary antibodies, followed by visualization of the protein bands. The antibody information was recorded in Table S3 (Supporting Information).

### Co‐Immunoprecipitation (co‐IP) Assay

Cells were lysed with IP Lysis Buffer (Thermo Fisher Scientific) for 5 min on ice. The lysate was centrifuged at 13 000 rpm for 10min. The supernatant was collected and added to Anti‐DYKDDDDK Magnetic Agarose (A36797, Thermo Fisher Scientific) pre‐washed with PBS, and incubated for 20 min. After incubation, the supernatant was discarded, and the agarose was washed twice with PBS. 100ul 5 × Laemmli buffer was added to agarose and then heated at 95 °C for 10 min. The samples were then subjected to a Western blot.

### FLAG Epitope Tag Knock‐In via CRISPR‐Cas9

sgRNA targeting the end of protein‐coding sequence of VPS72 gene was cloned into px459 vector for sgRNA and Cas9 expression. The neomycin resistant gene on pFETCh_Donor (EMM0021) (63 934, Addgene) was replaced by a Blasticidin (BSD) resistance gene, and the −800 and + 800 sequences flanking the stop codon of VPS72 were amplified by PCR and inserted into the upstream and downstream of FLAG‐FLAG‐FLAG‐BSD cassette respectively to serve as the template for homology directed repair. The modified px459 and pFETCh plasmids were transfected into Huh7 cells at a 2:1 mass ratio with GenJet transfection reagent. A control group was included with the modified px459 and original pFETCh plasmids. BSD selection at a concentration of 2.5ug/ml was conducted from 72 h post‐transfection and lasted for 14 days. Wild type Huh7 conditioned medium was used during BSD selection to support the growth of single cells, medium and BSD were replaced every 3 days. After BSD selection, the colonies that survived were transferred into 24‐well plates for amplification and genotyping. The cells were maintained in 1ug/ml BSD. For genotyping, partial of the cells were collected in a small volume of ddH2O and served as PCR templates. Cloning primer sequences of sgRNA were listed in Table S4 (Supporting Information). sgRNA sequences and locations were listed in Table S5 (Supporting Information).

### Gene Expression Regulation by CRISPR‐dCas9‐Based DNA Methylation

Two systems were used. The DNMT3A‐3L system is described before.^[^ ^49^ ^]^ Briefly, pAC1371‐pX‐sgRNA‐5xPBSa bearing gene‐specific sgRNA, px330‐dCas9, and pcDNA3‐PUFa‐Dnmt3A‐3L plasmids were co‐transfected into cells by ViaFect transfection reagent, and the gene expression levels were checked by qPCR. In another system, two lentiviruses were generated and co‐transduced into cells. The first lentivirus was generated with lentiGuide‐Puro plasmid bearing gene‐specific sgRNA. The second lentivirus was generated with pHAGE‐TRE‐dCas9‐KRAB plasmid with a MeCP2 coding sequence inserted after KRAB. Doxycycline was used to induce the expression of the dCas9‐KRAB‐MeCP2 fusion protein to catalyze DNA methylation near the sgRNA targets.

### Nile Red Staining Assay

For the staining, cells on 12‐well plates (Corning) with a cell density higher than 50% were utilized. Following the disposal of the growth media, the cells were rinsed with phosphate‐buffered saline (PBS) and fixed with 4% PFA for 10 min. Following fixation, cells were rinsed with ddH2O. Nile Red (Thermo) and Hoechest 33 342 solution (Dojindo Laboratories) were added into PBS following the manufacturers’ instructions to form a staining solution. Cells were stained with the solution for 5 min in dark. After staining, cells were washed with ddH2O and imaged using a BioTek Cytation1 Cell Imaging Multimode Reader. The fluorescence intensity was calculated with ImageJ.

### MspJI‐based DNA Methylation Assay

Genomic DNA was extracted from cells with the phenol‐chloroform method. 2ug of gDNA were digested with XmaJI to reduce its complexity. Digested gDNA were alcohol precipitated and reconstituted in ddH2O for cleanup. Then half of the digested gDNA were further digested with MspJI while the other half served as undigested control. After MspJI digestion, the enzyme was heat‐inactivated, and the reaction was diluted for RT‐PCR. 5 ng of gDNA were used for each reaction as template. A target sequence in gene desert that contains no XmaJI and MspJI digestion sites was used as the endogenous control, while the target sequences were designed to have MspJI cleavage sites inside but without XmaJI sites. The standard ΔΔCt method was used to calculate the percentage of the undigested (unmethylated) target sequence in the template compared to the undigested control of each sample.

### RNA‐seq

For RNA‐seq preparation, rRNA was depleted by using QIAseq FastSelect −rRNA HMR Kit (334 376, Qiagen), and the library was prepared by using KAPA RNA HyperPrep Kit (Roche). The libraries were sequenced with the Illumina NovaSeq 6000 platform by HaploX (Jiangxi, China).

### ChIP‐seq

The samples are prepared as previously described.^[^ ^50^ ^]^ Briefly, chromatin was sheared to fractions size centered at 200–250 bp by sonication with Covaris m220 sonicator. DNA fragments were incubated with target protein antibodies, and Pierce Protein A/G Magnet Beads were used to purify antibodies and the attached DNA fragments. DNA libraries were prepared by using NEBNext Ultra II DNA Library Prep Kit for Illumina and library size was selected by using Promega ProNex Size‐Selective Purification System. The samples were subjected to ChIP‐Seq by HaploX (Jiangxi, China).

### ATAC‐seq

50000 of nucleuses were incubated with Tagment Enzyme A50 from the Rapid DNA Lib Prep Kit (ABclonal) for DNA end preparation. The library was prepared by following PCR condition (65 °C, 5 min; 98 °C, 30 sec; 98 °C, 10 sec; 65 °C, 75 sec). Repeat steps 3–4, cycle rounds to be determined by parallel real‐time PCR. ATAC‐seq libraries were sequenced with Illumina HiSeq X Ten System, peak calling was done with MACS2.^[^ ^51^ ^]^

### Mouse Xenograft Model

The research received approval from the Committee on the Use of Live Animals in Teaching and Research (CULATR) from The University of Hong Kong (HKU, Approval No. 5712–21). The assay was conducted as previously described.^[^ ^48^ ^]^ Briefly, the Huh7 shVPS72 cells were resuspended in sterilized PBS, and injected subcutaneously into the flanks of female nude mice aged 4 to 6 weeks. The amount of injected cells was 5 × 10^6^ cells per mouse. The weight of the mice and the volume of their tumors were measured every three days. Five weeks after the injection, the mice were anesthetized using an intraperitoneal injection of pentobarbital and then euthanized by dissection. Tumor xenografts were then excised and weighed.

### Immunohistochemistry (IHC) Assay

IHC assay was performed as previously described.^[^ ^48^ ^]^ Briefly, tumor tissues were harvested, fixed in 4% PFA, embedded in paraffin, and sectioned to make slides. The slides were then stained with primary antibodies, secondary antibodies, DAB colorimetric solution, and Hematoxylin, scanned under a microscope, and photographed.

### Data Analysis

Data for HCC patients were obtained from cBioPortal, specifically from the Liver Hepatocellular Carcinoma (TCGA, PanCancer Atlas) dataset. Experimental results were derived from a minimum of three independent replicates. Statistical analyses were conducted using unpaired two‐tailed t‐tests, with data expressed as the mean ± standard deviation (S.D.). The following indicators were used to represent statistical significance: ^*^ *p* < 0.05, ^**^ *p* < 0.01, ^***^ *p* < 0.001, and ^****^ *p* < 0.0001.

## Conflict of Interest

The authors declare no conflict of interest.

## Author contributions

Q.Z. and Y.H. are co‐first authors and contributed equally to this work. JZ conceived the project, designed the experiments, and analyzed the data. Q.Z., Y.H. N.K, and Y.T. designed and performed the experiments and analyzed the data; Q.Z., Y.H., and J.Z. wrote the manuscript. All authors have read and agreed to the published version of the manuscript.

## Supporting information



Supporting Information

Supporting Information

## Data Availability

The data that support the findings of this study are available in the supplementary material of this article.
