# Supplementary material for: Copy Number Gains of VPS72 Drive De Novo Lipogenesis and Hepatocarcinogenesis via ATF3/mTORC1/SREBP1 Axis
Source: Adv Sci (Weinh). 2025 Apr 30;12(20):2411368. doi: 10.1002/advs.202411368 (PMC12120707; doi:10.1002/advs.202411368)
Supplement: Supplementary file 1 — Supporting Information [file ADVS-12-2411368-s002.docx]

Supporting Information

**Copy number gains of VPS72 drive de novo lipogenesis and hepatocarcinogenesis via ATF3/mTORC1/SREBP1 axis**

**Qinglin Zhang*, Yunxing Huang*, Yin Tong, Kenneth Tsz Chun Ng, Jiangwen Zhang^#^**

*: These authors contribute equally to the work. #: Corresponding Author.

**Figure S1**


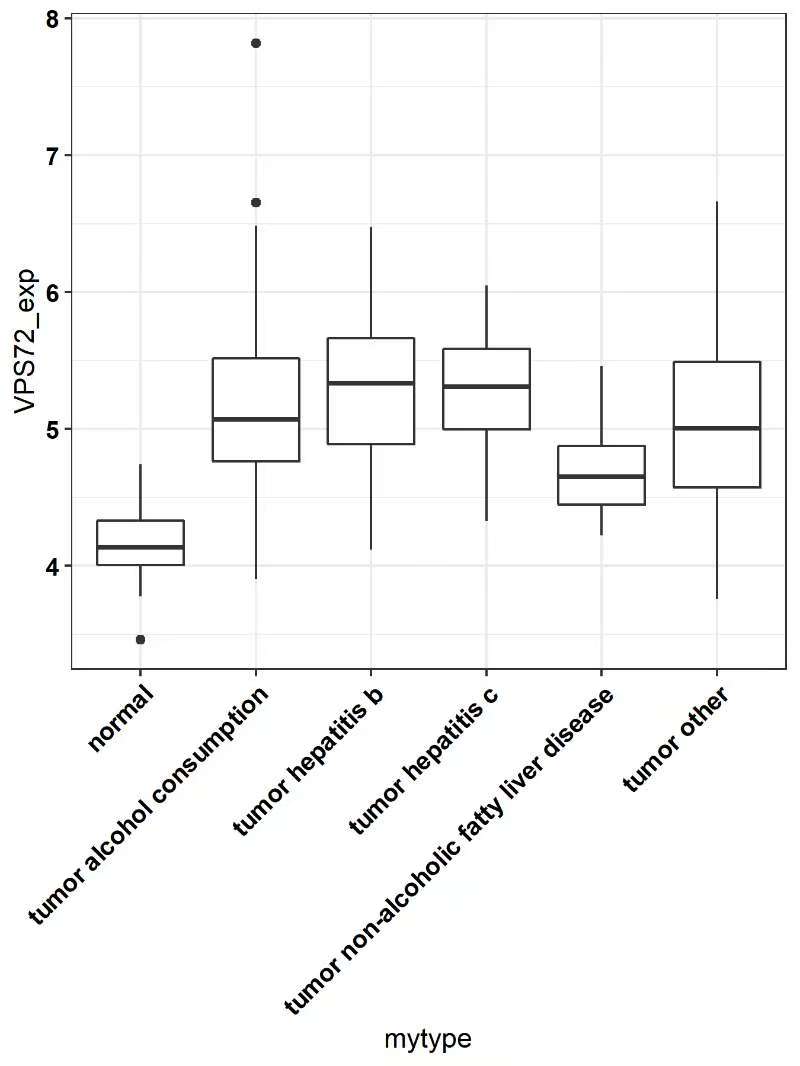
**Figure S1. VPS72 expression in normal patients and various types of HCC patients.**

**
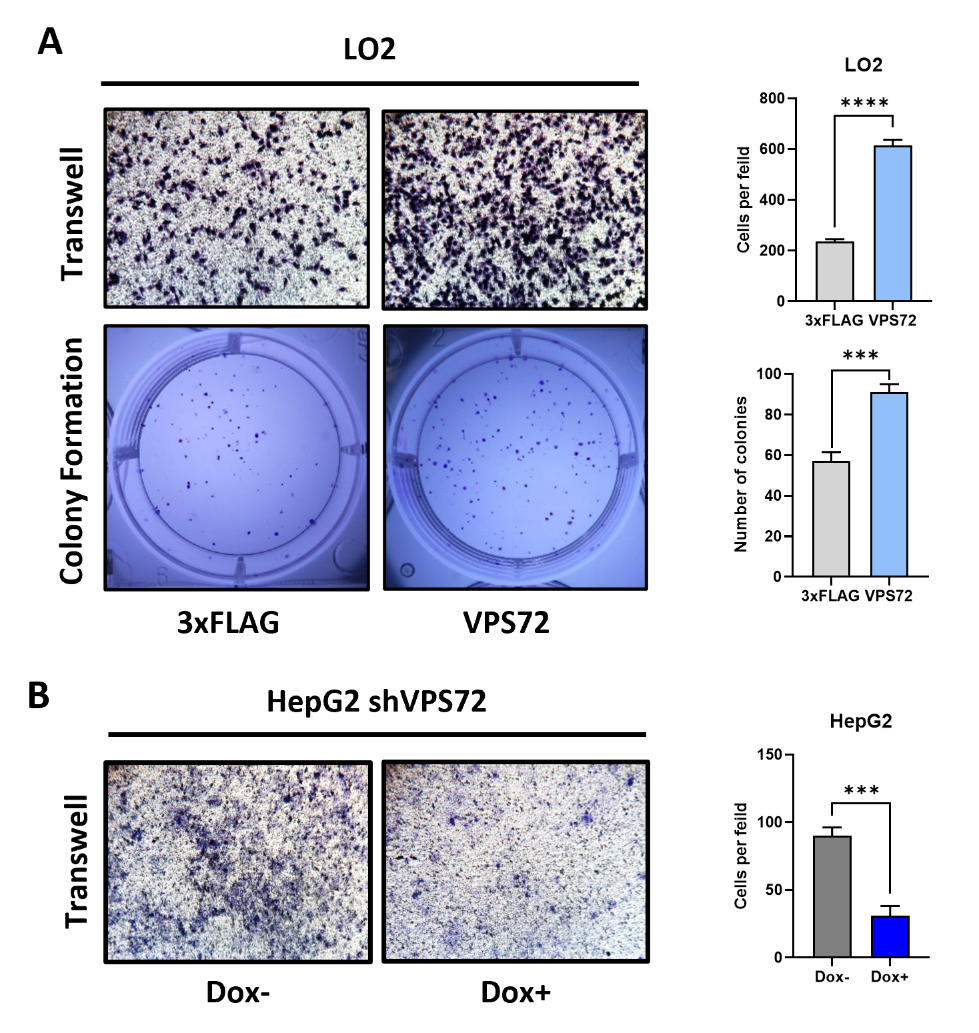
Figure S2**

**Figure S2. VPS72 promoted cell migration and proliferation ability.** (A) Transwell and colony formation assay in LO2 cells overexpressing VPS72. (B) Transwell assay in HepG2 cells with knockdown of VPS72. n=3 independent experiments. *** denotes p< 0.001 and **** denotes p< 0.0001.

**
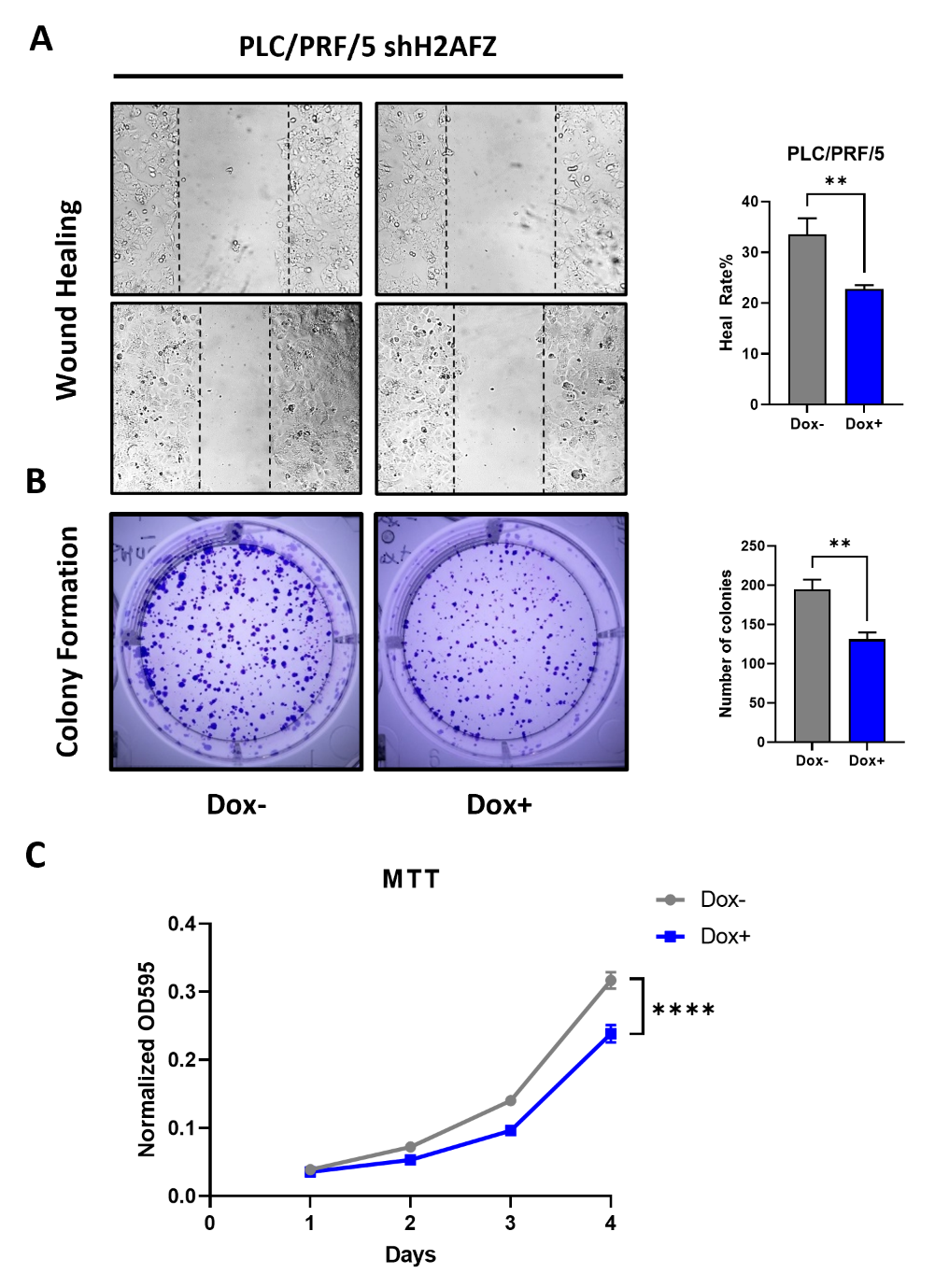
Figure S3**

**Figure S3. Knockdown of H2AFZ inhibited migration and proliferation of PLC/PRF/5 cells.** (A) Wound-healing assay. (B) Colony formation and (C) MTT assay. n=3 independent experiments. ** denotes p< 0.01 and **** denotes p< 0.0001.


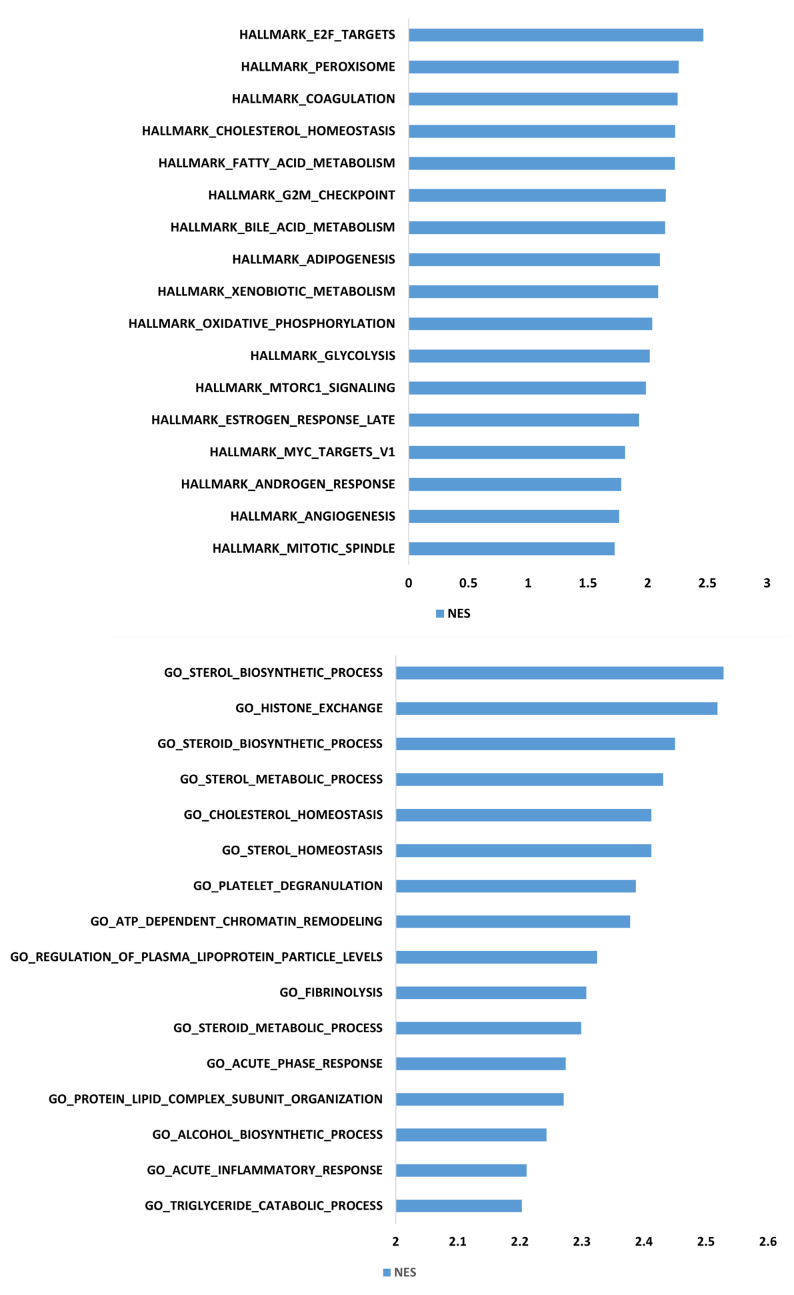
**Figure S4**

**Figure S4. GSEA gene ontology and hallmark analysis of RNA-seq results.**

**Figure S5**

**
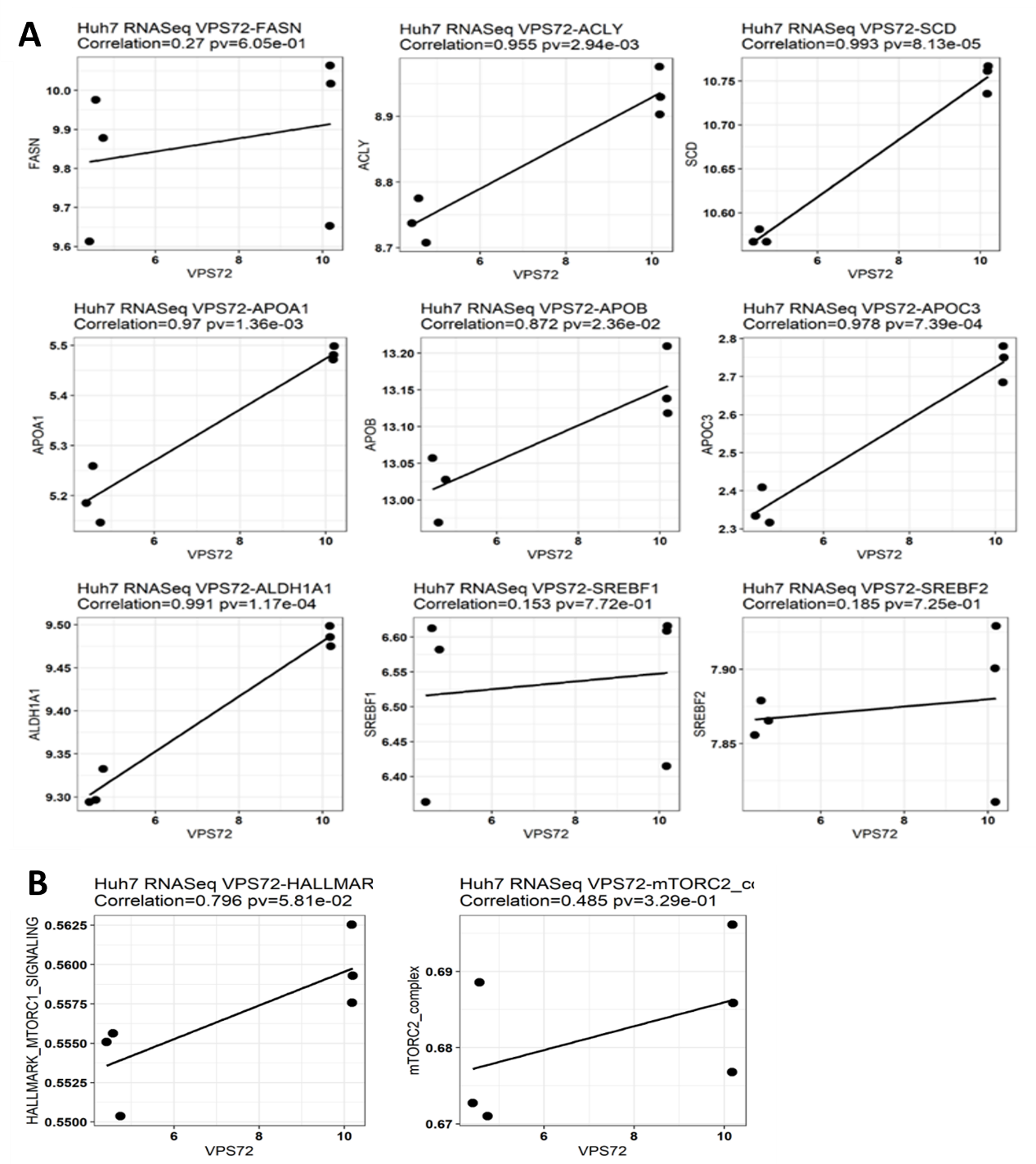
**

**Figure S5. Correlation analysis based on RNA-Seq result between VPS72 and key lipogenic genes, and mTORC1 activity upon overexpression of VPS72.** (A) Correlation analysis based on RNA-Seq result between VPS72 and key lipogenic and lipoprotein-associated genes (SREBF1, SREBF2, FASN, ACLY, SCD, ALDH1A1, APOA1, APOB, and APOC3). (B) Correlation analysis based on RNA-Seq result between VPS72 and mTORC1 signaling hallmark and mTORC2 complex components.

**Figure S6**

**
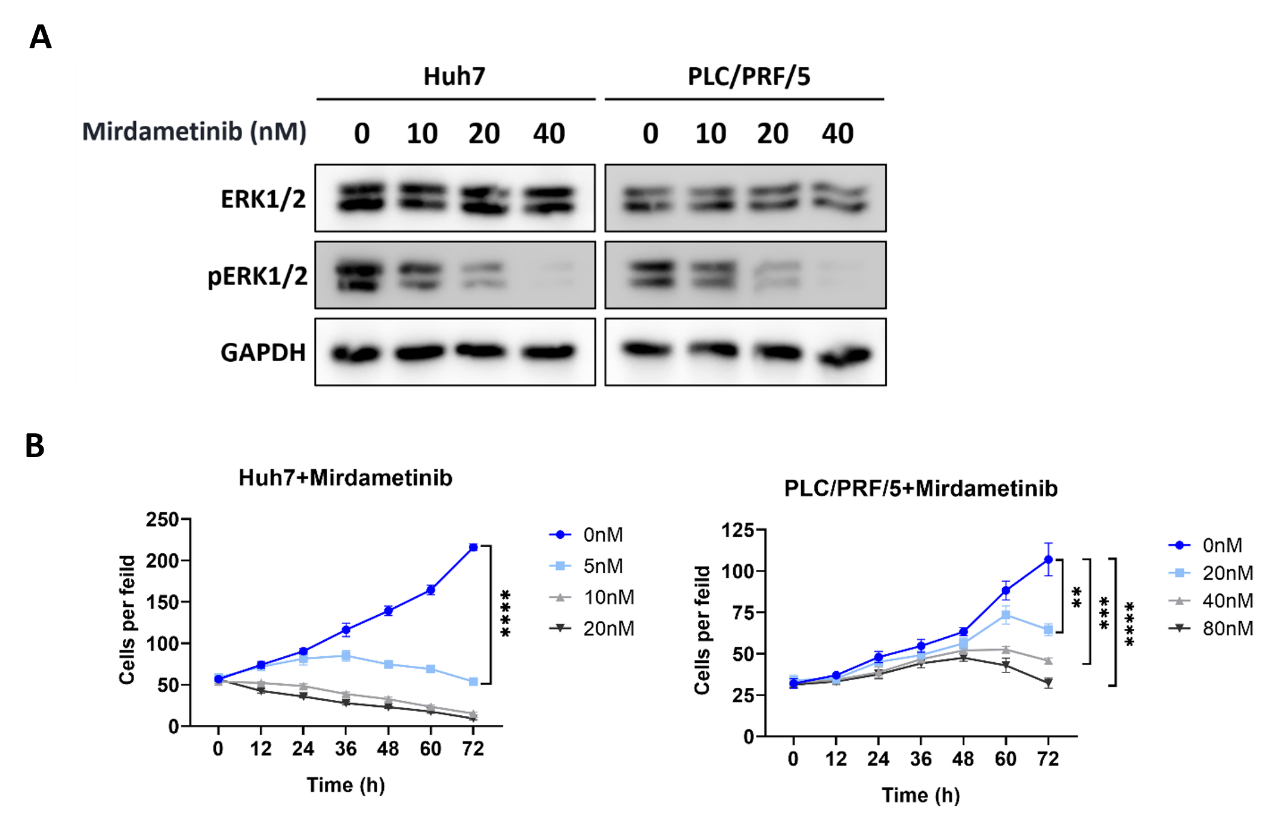
**

**Figure S6. Inhibition of ERK1/2 represses the proliferation of HCC cells.** (A) Western blot analysis of ERK1/2 and phosphorylated ERK1/2 protein levels in Huh7 and PLC/PRF/5 cell lines treated with various doses of ERK1/2 inhibitor mirdametinib for 24h. (B) Cell proliferation analysis of cell lines in (A). n=3 independent experiments. ** denotes p< 0.01, *** denotes p< 0.001 and **** denotes p< 0.0001.

**Figure S7**

**
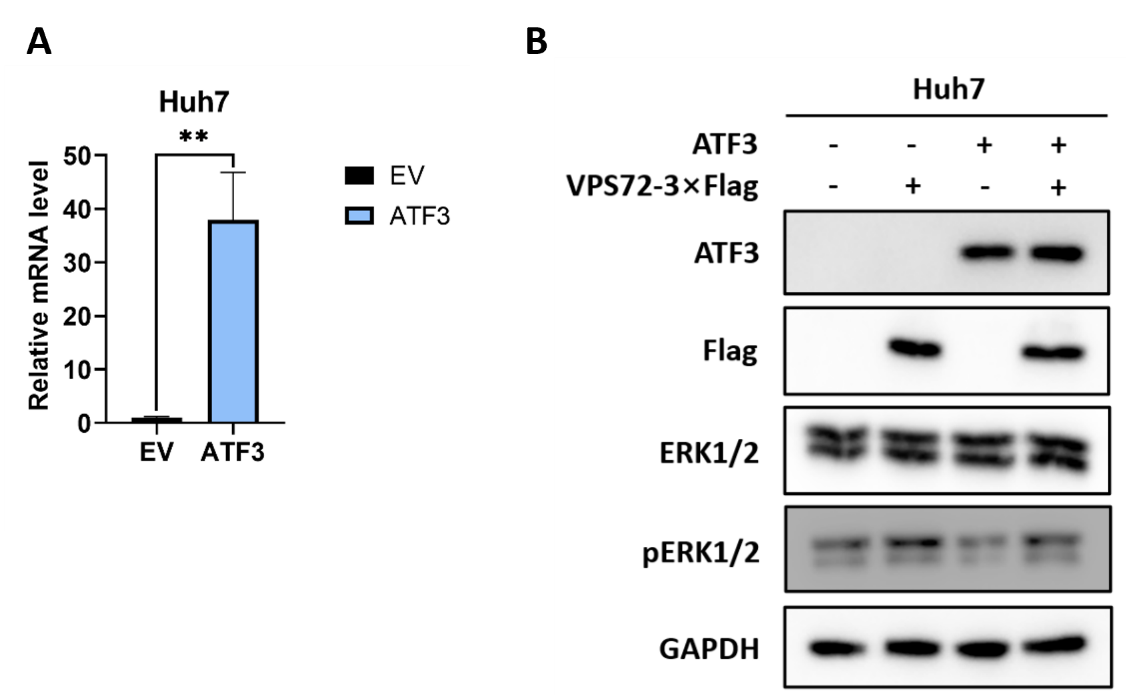
**

**Figure S7. Validation of ATF3 and VPS72-3×Flag expression in Huh7 cell line.** (A) RT-qPCR analysis of ATF3 mRNA levels in Huh7 cells transfected with an empty vector or ATF3 overexpression construct. ATF3 mRNA expression increased by over 30-fold upon ATF3 overexpression. (B) Western blot analysis of VPS72, ATF3, ERK1/2 and phosphorylated ERK1/2 protein levels in Huh7 cells transfected with VPS72-3×Flag, ATF3, or both, compared to the empty vector controls. These cells were used for analysis in Fig 5J and Fig 5L. Basal ATF3 protein expression was undetectable in control cells due to the robust signal in the ATF3-overexpressing group. n=3 independent experiments. ** denotes p< 0.01.

**Table S1**

**Table S1. Sequences of primers for cloning**

| **cDNA Cloning for Viral Expression** | |
| --- | --- |
| VPS72-F | ACCATCTCGGTAGGCGGTATGAGTTTGG |
| VPS72-R | AGCCTGATTTAATGACAATTTTCTGGCGCAAGGC |
| ATF3-F | GACCGGTTCTAGAGTGCTGCCACCATGATGCTTCAACAC |
| ATF3-R | AGTCTGTACCTCCGCTGCCTGAGCCGCTCTGCAATGTTCCTTCTTTTA |
| SIRT7-F | ACCATGGCAGCCGGGG |
| SIRT7-R | AGCCTGACGTCACTTTCTTCCTTTTTGTGC |
| Nrf2-F | ACCATGATGGACTTGGAGCTG |
| Nrf2-R | AGCCTGAGTTTTTCTTAACATCTGGCTTCTTAC |
| **VPS72 Homology Arms Amplification for CRISPR-KI** | |
| Flanking-F | CAGTCACTTAGTACGTTCCTCAG |
| Flanking-R | AGATACGCATAAGGAGCTATGC |
| HA1-F | TCCCCGACCTGCAGCCCAGCTAATGACAATTTTCTGGCGCAAGGCCAACACCCTGCCTCC |
| HA1-R | CCGGAACCTCCTCCGCTCCCTTTAATGACAATTTTCTGGCGCAAtGCTCGGGGCCCAG |
| HA2-F | GGAGGGCTAATTCGAACATCGCCAGAAAATTGTCATTAAATGAAG |
| HA2-R | TGGAGAGGACTTTCCAAGCCTTGCGCCAGAAAATTGTCATTCACTGAGTGACAGAGACAG |
| **pFETCh Vector Replace Neo with Bsd** | |
| pFET-F | GCGAAACGATCCAGGTCC |
| pFET-R | TTCGAACATCATGTCTTCTTATACGAAG |
| pFET-Bsd-F | ACCTGGATCGTTTCGCATGGCCAAGCCTTTGTCTC |
| pFET-Bsd-R | GAAGACATGATGTTCGAATTAGCCCTCCCACACATAACC |
| **Remove BbsI Site Inside Bsd in pFETCh-BSD Vector** | |
| BSDmut-F | GACGCTGTAATCCTCAGAGATGGGGATGCTGTTGATTGTAGCC |
| BSDmut-R | CTCTGAGGATTACAGCGTCGCCAGCGCAGCTCTCTCTAG |
| **Genotyping of VPS72 CRISPR-KI** | |
| colPCR-F | CAAGGATGTGTTTTGGTGTGC |
| colPCR-R | AAGTACATTACTGCCCATGG |
| **Target Gene** | **shRNA (With sticky ends for cloning)** |
| VPS72 | AAAATCCGGAGATGAGTATCAATTGGATCCAATTGATACTCATCATCTCCGGA |
| H2AFZ | AAAACCGTATTCATCGACACCTAAATTGGATCCAATTTAGGTGTCGATGAATACGG |
| SIRT7 | AAAACACCTTTCTGTGAGAACGGAATTGGATCCAATTCCGTTCTCACAGAAAGGTG |
| SIRT1 | AAAACAGGTCAAGGGATGGTATTTATTGGATCCAATAAATACCATCCCTTGACCTG |

**Table S2**

**Table S2. Sequences of RT-qPCR primers**

| **RT-PCR Primers for Gene Expression Level** | | |
| --- | --- | --- |
| **Gene** | **Forward** | **Reverse** |
| GAPDH | GATGACATCAAGAAGGTGGTGAA | GTCTTACTCCTTGGAGGCCATGT |
| PPIA | TATGTGTCAGGGTGGTGAC | CGTATGCTTTAGGATGAAGTTCTC |
| RPL13A | AAGTACCAGGCAGTGACAG | TTTCCGTAGCCTCATGAGC |
| VPS72 | TTCACAGAGGAATCCGGAG | AATGTCAAAGTCAGAGTCCAC |
| H2AFZ | TAGGACGACCAGTCATGGA | TTCAAGTACCTCTGCGGTG |
| ATF3 | ATCAGTCACTGTCAGCGAC | TCTCCGACTCTTTCTGCAG |
| ACACB | CATTAGCTTGGTGACCTGC | TTCTCCACCTGGATCACTC |
| FASN | CAACCTCTCCCAGGTATGC | TGCTGATGATGGACTCCAG |
| ACLY | AAGTATGCTCGGGTCACTC | TACCAAGTTCTGGCTGAGC |
| SCD1 | CACCGCTCTTACAAAGCTC | ATAGACATCATTCTGGAATGCC |
| ALDH1A1 | GTGGCAAGAAATTTCCTGTC | TCAACATCCTCCTTATCTCCT |
| SREBF1 | TCTCTTAGAGCGAGCACTG | TCCGAGAATTCCTTGTCCC |
| SREBF2 | GATGCAAAGGTCAAAGATGAG | TTAAAGGAGAGGCACAGGA |
| APOA1 | CTTCAGGATGAAAGCTGCG | AGTTGTCAAGGAGCTTTAGG |
| APOA2 | AGACCGTGACTGACTATGG | CAAAGTAAGACTTGGCCTCG |
| APOB | CTGATGGTGTCTCTAAGGTC | TTCCATTTACCATATCCTGCTC |
| APOC3 | GTTACATGAAGCACGCCAC | TTTCAGGGAACTGAAGCCA |
| CCDC106 | TCA TTG TGG CCC CCG AGA A | TTG AAG CGG TAG GTG ATG GG |
| GADD45B | TGG TTG GTC CTT GTC TGC A | GCT TCC CAT CTC GCT CTC A |
| KLF10 | AAG GAG GTT TGC CCG TTC TG | AGC AGG GGT TGG AGG TAG AG |
| LIG4 | AGC TGC CCC AAA GAT GAA GA | TGT CTG GGC CTG GAT TTT GT |
| PLD6 | ACG AGT ACG TGC GGC TTT T | TTT GGC TTT CGC TGG AGG T |
| SLC7A11 | GGTGGAACGAGGAGGTGGAG | TGTGCTTTTTCCTTCACAGCGA |
| TIPARP | AGT GGG ACG GTG GCA GAT T | AAC ACG AGG TCA AGG GAA GC |
| TNFAIP3 | TGC ACC GAT ACA CAC TGG AA | CCG TCA CCG TTC GTT TTC A |
| TRMT61B | TCA GGA GCA ACC GAA GAC ATA | GCA AGG CAA ACC AAC CAA TC |
| ZIC5 | CAG CAA CGG AAC CAC CTC T | GTC TCA GGC TCG GCA TTG T |
| **RT-PCR Primers for MspJI DNA Methylation Assay** | | |
| CRE | ATTTCACGGTTCTCCTGGGG | AGCTGTTCACGGAAGTCTGA |
| CpG1 | TCATTCAAATGCAAACACTCCTCC | TAAACCCCGGGCGTGTCG |
| CpG2 | CCGTTACCAGGGCGAAAAGT | ACCTGAACCGATTTCCAGCG |
| CpG3 | AGAAATTGTTGGGGTCGGGG | GTGGTCCGAGATTCGAGCTG |
| CpG4 | CCGCTTTTGTGTTAACCGGC | GGGCAGAAGCGTTAGTCTGT |
| Pro | TGAGGGCTATAAAAGGGGTGATGC | GCGAGAGAAGAGAGCTGTGCAGTG |
| **RT-PCR Primers for ChIP-qPCR** | | |
| ChIP-GADD45B-1 | CGATCCGATGGAGAAGGGGG | GACCCTCCGTGATGGGGAAG |
| ChIP-PLD6-1 | TCGCATGGAGCACAGCAGAG | AAAACCTCTGGGCCGACACC |
| ChIP-PLD6-2 | CAGCATACTGGAGGCGGTGT | CCTCGGCTGCCTTAGTCTCT |
| ChIP-ATF3-1 | GGGTGGTCTGAGTGAGGTCG | GGCCGAAGGTCACAGTTTGG |
| ChIP-ATF3-2 | CGCCGCTGGAAATCGGTTCA | CAAGCCCGACCTCACTCAGAC |
| ChIP-H3K27ac-GADD45B-1 | GCTTTCCGCACGCTTGTCTT | ACATCAGGATACGGCAGCCC |
| ChIP-H3K27ac-GADD45B-2 | GGTCTCAGGATGCAGACGGT | TCCATGCAAGACAAGCGTGC |
| ChIP-H3K27ac-PLD6-1 | TGCAAGGTCACGCCCTCAG | TCACCTGTTGAGGGTTGGGG |
| ChIP-H3K27ac-PLD6-2 | CGCTAGAAGCCACAGCAACC | ACAGTGAAGGTGTGACCCCA |

**Table S3**

**Table S3. Information of antibodies**

| **Target** | **Ref** | **Company** |
| --- | --- | --- |
| GAPDH | sc-47724 | Santa Cruz |
| FLAG | F3165 | Sigma |
| FLAG | 14793S | CST |
| FLAG (magnetic beads) | A36797 | Thermo Fisher |
| VPS72 | A303-115A | BETHYL |
| VPS72 | 15143-1-AP | ProteinTech |
| H2A.Z | PA5-21923 | Thermo Fisher |
| H2A.Zac | ab18262 | Abcam |
| H3K27ac | 8173S | CST |
| H3K4me1 | 5326S | CST |
| ATF3 | sc-81189 | Santa Cruz |
| totalS6K | sc-8418 | Santa Cruz |
| pS6K | sc-8416 | Santa Cruz |
| Anti-Rabbit-Dylight 650 | 84546 | Thermo Fisher |
| Anti-Mouse-Alexa 488 | A21202 | Thermo Fisher |
| Anti-Rabbit-HRP | sc-2357 | Santa Cruz |
| Anti-Mouse-HRP | 31430 | Thermo Fisher |
| Anti-Sheep-HRP | 61-8620 | Thermo Fisher |
| Anti-Rabbit-Lightchain-HRP | 211-032-171 | JacksonImmunoResearch |
| H2A.Z | 16441-1-AP | ProteinTech |
| FASN | 10624-2-AP | ProteinTech |
| ACLY | 15421-1-AP | ProteinTech |
| SCD | 28678-1-AP | ProteinTech |
| ALDH1A1 | 15910-1-AP | ProteinTech |
| SREBF1 | 14088-1-AP | ProteinTech |
| mTOR | 66888-1-Ig | ProteinTech |
| AMPK | 10929-2-AP | ProteinTech |
| p-AMPK | 50081 | CST |
| ERK1/2 | 11257-1-AP | ProteinTech |
| p-ERK1/2 | 80031-1-RR | ProteinTech |

**Table S4**

**Table S4. Sequences of cloning primers of sgRNA**

| **sgRNA for VPS72-CRISPR-KI** | |
| --- | --- |
| sg-F | CACCGAATGACAATTTTCTGGCGCA |
| sg-R | AAACTGCGCCAGAAAATTGTCATTC |
| **sgRNA for ATF3 dCas9-DNMT3A-L** | |
| sg1-F | CACCGGGTTAATCCTCAATGAAAG |
| sg1-R | AAACCTTTCATTGAGGATTAACCC |
| sg2-F | CACCGTGTGCACACTGGACATTGG |
| sg2-R | AAACCCAATGTCCAGTGTGCACAC |
| sg3-F | CACCGCTTGAATGAAACTCTTACCC |
| sg3-R | AAACGGGTAAGAGTTTCATTCAAGC |
| sg4-F | CACCGTAATAAGAATAACCCGACCG |
| sg4-R | AAACCGGTCGGGTTATTCTTATTAC |
| sg5-F | CACCGAAGCCCCCAACACCCCGGT |
| sg5-R | AAACACCGGGGTGTTGGGGGCTTC |
| sg6-F | CACCGCACCATTATAAAGTGAAGCA |
| sg6-R | AAACTGCTTCACTTTATAATGGTGC |

**Table S5**

**Table S5. Sequences and location of sgRNA**

| **Name** | **Sequence** | **Location** | **Location to ATF3 promoter** |
| --- | --- | --- | --- |
| sg1 | GGGTTAATCCTCAATGAAAG | 212,605,886-212,605,905 | -1102~-1083 |
| sg2 | GTGTGCACACTGGACATTGG | 212,606,038-212,606,057 | -950~-931 |
| sg3 | GCTTGAATGAAACTCTTACCC | 212,606,247-212,606,266 | -741~-722 |
| sg4 | GTAATAAGAATAACCCGACCG | 212,606,434-212,606,453 | -554~-535 |
| sg5 | GAAGCCCCCAACACCCCGGT | 212,606,451-212,606,470 | -537~-518 |
| sg6 | GCACCATTATAAAGTGAAGCA | 212,606,696-212,606,715 | -292~-273 |

**Table S6**

**Table S6. Top 30 DEGs of RNA-Seq data**

| **symbol** | **logFC** | **AveExpr** | **t** | **P.Value** | **adj.P.Val** | **B** | **VPS72_OE_avg** | **FLAG_avg** | **median_difference** | |
| --- | --- | --- | --- | --- | --- | --- | --- | --- | --- | --- |
| VPS72 | 5.606504 | 7.380168 | 62.62508 | 4.25E-09 | 5.70E-05 | 3.677589 | 10.18342 | 4.576916 | 5.613317 | 1162.827 |
| LINC02444 | -1.13859 | 0.019441 | -11.1695 | 5.30E-05 | 0.278349 | 1.791614 | -0.54985 | 0.588734 | -1.14928 | 0.68309 |
| SLC7A11 | -0.47657 | 5.680816 | -10.7047 | 6.64E-05 | 0.278349 | 1.668555 | 5.442529 | 5.919103 | -0.47191 | 43.4875 |
| ATF3 | -0.84635 | 4.600304 | -9.92689 | 9.90E-05 | 0.278349 | 1.438232 | 4.17713 | 5.023477 | -0.79021 | 18.09012 |
| ALOXE3 | -2.19675 | -1.03891 | -9.08527 | 0.000158 | 0.278349 | 1.148994 | -2.13728 | 0.059462 | -2.37947 | 0.227307 |
| LIG4 | -0.8753 | 3.753007 | -9.06561 | 0.000159 | 0.278349 | 1.141678 | 3.315359 | 4.190656 | -0.97112 | 9.954567 |
| TNFAIP3 | -0.67616 | 5.084641 | -9.03242 | 0.000163 | 0.278349 | 1.129267 | 4.746561 | 5.422721 | -0.62523 | 26.84462 |
| FAM81A | 0.768202 | 1.2523 | 8.832755 | 0.000183 | 0.278349 | 1.052927 | 1.636401 | 0.868199 | 0.696721 | 3.108894 |
| ZIC5 | -0.40863 | 2.407266 | -8.79642 | 0.000187 | 0.278349 | 1.038719 | 2.202951 | 2.611581 | -0.42331 | 4.604202 |
| ANO9 | -1.50737 | 1.786802 | -8.59828 | 0.00021 | 0.281625 | 0.95949 | 1.033118 | 2.540487 | -1.37357 | 2.046442 |
| ATP2A1 | -0.54012 | 0.486564 | -8.38208 | 0.00024 | 0.281625 | 0.869535 | 0.216505 | 0.756624 | -0.58708 | 1.161915 |
| ROCK1P1 | -1.21979 | 0.422973 | -8.19872 | 0.000269 | 0.281625 | 0.790248 | -0.18692 | 1.032867 | -1.16045 | 0.878479 |
| ADGRF4 | -1.01731 | 1.057123 | -8.17583 | 0.000273 | 0.281625 | 0.780153 | 0.54847 | 1.565775 | -0.86275 | 1.462534 |
| NEBL | -0.89079 | 0.989304 | -7.99361 | 0.000306 | 0.291472 | 0.698148 | 0.543907 | 1.434701 | -0.85799 | 1.457915 |
| GRIK2 | 0.488895 | 2.269212 | 7.898908 | 0.000326 | 0.291472 | 0.654367 | 2.51366 | 2.024765 | 0.435746 | 5.710669 |
| ASTN2 | -0.39043 | 1.382488 | -7.52104 | 0.000419 | 0.292759 | 0.471347 | 1.187273 | 1.577703 | -0.36293 | 2.277218 |
| APOC3 | 0.385113 | 2.545702 | 7.517995 | 0.00042 | 0.292759 | 0.469815 | 2.738259 | 2.353145 | 0.416311 | 6.672645 |
| FGFR1 | -0.596 | 3.387449 | -7.36104 | 0.000467 | 0.292759 | 0.389643 | 3.089449 | 3.685449 | -0.61906 | 8.511709 |
| KLF10 | -0.45429 | 5.932199 | -7.19062 | 0.000526 | 0.292759 | 0.299723 | 5.705053 | 6.159345 | -0.48281 | 52.16656 |
| ADM5 | -0.62967 | 1.499155 | -7.1431 | 0.000544 | 0.292759 | 0.274098 | 1.184322 | 1.813988 | -0.56403 | 2.272566 |
| RNU6-26P | -1.04942 | -0.41795 | -7.10982 | 0.000557 | 0.292759 | 0.256013 | -0.94265 | 0.106763 | -1.07315 | 0.520275 |
| SOCS3 | -0.34106 | 2.730729 | -7.0913 | 0.000564 | 0.292759 | 0.245894 | 2.5602 | 2.901257 | -0.32648 | 5.897895 |
| CA12 | 0.428754 | 4.661257 | 7.016851 | 0.000595 | 0.292759 | 0.204836 | 4.875634 | 4.44688 | 0.458438 | 29.35703 |
| NOTCH2NLC | -0.67207 | 0.946916 | -6.96717 | 0.000617 | 0.292759 | 0.177098 | 0.610883 | 1.282949 | -0.72609 | 1.527194 |
| PLD6 | -0.38942 | 2.487125 | -6.95201 | 0.000624 | 0.292759 | 0.168579 | 2.292414 | 2.681836 | -0.37127 | 4.898752 |
| TIPARP | -0.30937 | 7.21393 | -6.8576 | 0.000668 | 0.292759 | 0.114936 | 7.059245 | 7.368615 | -0.31306 | 133.3658 |
| IFT46 | -0.46674 | 2.256452 | -6.85698 | 0.000668 | 0.292759 | 0.114583 | 2.023081 | 2.489822 | -0.45925 | 4.06451 |
| CCDC106 | -0.76151 | 1.811184 | -6.85376 | 0.00067 | 0.292759 | 0.112733 | 1.430427 | 2.191941 | -0.73438 | 2.695265 |
| GADD45B | -0.38309 | 5.49516 | -6.82914 | 0.000682 | 0.292759 | 0.098569 | 5.303614 | 5.686705 | -0.33664 | 39.49543 |
| MT1M | -1.43682 | 0.092102 | -6.76427 | 0.000716 | 0.292759 | 0.060906 | -0.62631 | 0.810514 | -1.50227 | 0.647831 |
| TRMT61B | -0.43367 | 3.363375 | -6.72455 | 0.000737 | 0.292759 | 0.037597 | 3.146541 | 3.580209 | -0.42297 | 8.855298 |
